# Past climate changes, population dynamics and the origin of Bison in Europe

**DOI:** 10.1186/s12915-016-0317-7

**Published:** 2016-10-21

**Authors:** Diyendo Massilani, Silvia Guimaraes, Jean-Philip Brugal, E. Andrew Bennett, Malgorzata Tokarska, Rose-Marie Arbogast, Gennady Baryshnikov, Gennady Boeskorov, Jean-Christophe Castel, Sergey Davydov, Stéphane Madelaine, Olivier Putelat, Natalia N. Spasskaya, Hans-Peter Uerpmann, Thierry Grange, Eva-Maria Geigl

**Affiliations:** 1 Institut Jacques Monod, UMR7592, CNRS, University Paris Diderot, Epigenome and Paleogenome group, 15 rue Hélène Brion, 75013 Paris, France; 2 CNRS, USR 3336 IFRA (Institut Français de Recherche en Afrique), Nairobi, Kenya; 3 Aix-Marseille Université, UMR 7269 LAMPEA (Labo.Méd.de Préhistoire, Europe-Afrique) Maison Méditerranéenne des Sciences de l’Homme, BP 674, 13094 Aix-en-Provence, cedex 2, France; 4 Mammal Research Institute Polish Academy of Sciences, Genetics and Evolution Department, Waszkiewicza 1, 17-230 Bialowieza, Poland; 5 CNRS/UMR 7044/MISHA, 5 allée du Général Rouvillois, 67083 Strasbourg, France; 6 Zoological Institute, Russian Academy of Sciences, 199034 Saint Petersburg, Russia; 7 Diamond and Precious Metal Geology Institute of the Siberian Branch of the RAS, Yakutsk, Russia; 8 Muséum d’histoire naturelle de Genève (MHN), Département d’Archéozoologie, Route de Malagnou 1, 1208 Geneva, Switzerland; 9 North-East Science Station, Pacific Institute of Geography, Far East Branch, Russ. Ac. Sci., 678830 Cherskiy, Russia; 10 Musée national de Préhistoire, 24620 Les Eyzies de Tayac-Sireuil, France; 11 Archéologie Alsace, 11 rue Jean-François Champollion, 67600 Sélestat, France; 12 UMR 7041 ArScan - Archéologies environnementales - Maison de l’Archéologie et de l’Ethnologie, 92023 Nanterre, France; 13 Zoological Museum of Moscow Lomonosow, State University, Bolshaya Nikitskaya Str. 6, Moscow, 125009 Russia; 14 Institut für Ur- und Frühgeschichte und Archäologie des Mittelalters, Abteilung Ältere Urgeschichte und Quartärökologie, Zentrum für Naturwissenschaftliche Archäologie, Rümelinstr. 23, 72070 Tübingen, Germany

**Keywords:** Ancient DNA, Bison, Population dynamics, Evolution, Climate, Paleoenvironment, Next generation sequencing, Sequence capture

## Abstract

**Background:**

Climatic and environmental fluctuations as well as anthropogenic pressure have led to the extinction of much of Europe’s megafauna. The European bison or wisent (*Bison bonasus*), one of the last wild European large mammals, narrowly escaped extinction at the onset of the 20th century owing to hunting and habitat fragmentation. Little is known, however, about its origin, evolutionary history and population dynamics during the Pleistocene.

**Results:**

Through ancient DNA analysis we show that the emblematic European bison has experienced several waves of population expansion, contraction, and extinction during the last 50,000 years in Europe, culminating in a major reduction of genetic diversity during the Holocene. Fifty-seven complete and partial ancient mitogenomes from throughout Europe, the Caucasus, and Siberia reveal that three populations of wisent (*Bison bonasus*) and steppe bison (*B. priscus*) alternately occupied Western Europe, correlating with climate-induced environmental changes. The Late Pleistocene European steppe bison originated from northern Eurasia, whereas the modern wisent population emerged from a refuge in the southern Caucasus after the last glacial maximum. A population overlap during a transition period is reflected in ca. 36,000-year-old paintings in the French Chauvet cave. Bayesian analyses of these complete ancient mitogenomes yielded new dates of the various branching events during the evolution of *Bison* and its radiation with *Bos*, which lead us to propose that the genetic affiliation between the wisent and cattle mitogenomes result from incomplete lineage sorting rather than post-speciation gene flow.

**Conclusion:**

The paleogenetic analysis of bison remains from the last 50,000 years reveals the influence of climate changes on the dynamics of the various bison populations in Europe, only one of which survived into the Holocene, where it experienced severe reductions in its genetic diversity. The time depth and geographical scope of this study enables us to propose temperate Western Europe as a suitable biotope for the wisent compatible with its reintroduction.

**Electronic supplementary material:**

The online version of this article (doi:10.1186/s12915-016-0317-7) contains supplementary material, which is available to authorized users.

## Background

Drastic climatic fluctuations during the Pleistocene in the northern hemisphere led to population contractions, extinctions, re-expansions, and colonizations of fauna and flora [1]. Bison, along with other large ungulates, thrived during the middle and late Pleistocene [2]. Numerous cave paintings and engravings in France and Spain, such as those in the caves of Chauvet, Lascaux, and Altamira, attest to the important role this impressive animal played for the late Paleolithic hunter-gatherers. The steppe bison (*Bison priscus* (Bojanus, 1827)) appears in the fossil record during the early middle Pleistocene, replacing another archaic but smaller forest-adapted bison (*B. schoetensacki* [3]), which went extinct ca. 700 kiloyears ago (kya) [4]. Since *B. priscus* was adapted to the cold tundra-steppe, occurrence of its remains is considered indicative of open environments [5]. It roamed over Europe and Asia, and also crossed the Bering Strait during the middle Pleistocene to populate North America, where it evolved into the American Bison *B. bison* [3, 6, 7]. The numerous fossil remains display a pronounced sexual dimorphism, and a large initial body size, gradually decreasing throughout the Pleistocene [5]. Differences in morphology related to climatic, environmental, and topographic conditions have led several authors to propose a high diversity for the Pleistocene cold-steppe bison expressed as subspecies or ecomorphotypes [5, 6].

The taxonomy, evolutionary history, and paleobiogeography of the genus *Bison* in Eurasia, and of the European bison or wisent *B. bonasus* (Linnaeus, 1758) in particular, is still patchy despite a rich fossil record and its current endangered status (e.g., [8–11]). Indeed, two opposing hypotheses on the evolution of bison in Eurasia coexist [2]. Traditionally, it has been considered that bison developed within one single phylogenetic line (*B. schoetensacki – B. priscus – B. bonasus*), but it has also been proposed that at least two parallel lines of bison existed, one being the line of forest bison from *B. schoetensacki* (Freudenberg, 1910) to the recent *B. bonasus* and the other being the line of the steppe bison *B. priscus* (for a review see [2]). Thus, the phyletic relationships between *B. schoetensacki*, *B. priscus*, and *B. bonasus*, as well as the approximate date and geographical origin of the wisent, remain elusive, due in part to the limited power of paleontological studies to resolve species-level mammalian taxonomy issues or to detect broad-scale genetic transitions at the population level [12].

*B. priscus* disappeared from the fossil record of Western Europe at the end of the Pleistocene, around 12–10 kya, and relict populations of *B. priscus* seem to have survived until the beginning of the middle Holocene (7–6 kya) in Siberia (e.g., [13, 14]). In Europe, *B. priscus* is believed to have been replaced at the end of the Pleistocene or during the Holocene by the morphologically (eidonomically) distinguishable wisent *B. bonasus* [2, 10, 15, 16]. At least two sub-species are recognized: (1) *B. b. bonasus* Linnaeus, 1758 from the Lithuanian lowland and the Polish Białowieża ecosystem, and (2) the Caucasian highland *B. b. caucasicus* (Turkin and Satunin, 1904) [17]. *B. priscus* was adapted to forest-steppe and steppe, and *B. bonasus* to forest and mountain-forest environments. *B. priscus* and *B. bonasus* are anatomically much closer to each other than to other more ancient bison, such as *B. schoetensacki. B. bonasus* has a relatively more massive rear quarter and shorter horns compared to *B. priscus*, which has longer and slightly curved horns and a smooth double-humped appearance [15, 16]. *B. priscus* and *B. bison* (Linnaeus, 1758), both of which are grazers, have a lower head position than *B. bonasus*, which is a mixed feeder [18]. It is, however, very difficult to assign fossil bison bones to either species [2]. Paleolithic paintings of bison from caves in France and Spain are often classified as belonging to either *B. priscus* or *B. bonasus* [19]. The diversity of the cave art depictions and the large range of their occurrence is interpreted as indicating an origin of *B. bonasus* in the area between southern Europe and the Middle East and of its existence well before the end of the late Pleistocene at a time when *B. priscus* was still present [19].

Both extant bison species narrowly escaped extinction. The American *B. bison* was almost wiped out during the 19th century through commercial hunting and slaughter, but also due to introduced bovine diseases and competition with domestic livestock [20]. The wisent also almost went extinct at the beginning of the 20th century. Indeed, similar to other large herbivores, such as the aurochs, intensification of agriculture since the Neolithic period pushed the wisent into the forests of Eastern Europe [18], where it was strictly protected for several centuries as royal game [11]. During the First World War, however, a diminished population size followed by poaching led to its extinction in the wild [11]. The entire population living today is descended from just 12 out of the 54 surviving animals at the beginning of the 1920s [11].

The wisent is still poorly characterized genetically. While genetic markers from the autosomes and Y chromosomes of American bison and wisent are closer to each other than to the other members of the genus *Bos* and they can reproduce and give rise to fertile offspring, their mitochondrial genomes are phylogenetically separated [9, 21, 22]. Indeed, mitochondrial sequences of the American bison and the yak *Poephagus mutus* f. *grunniens* (Linnaeus, 1758) form a distinct cluster, while the wisent occupies a phylogenetic position closer to *Bos primigenius* f. *taurus* (Linnaeus, 1758), a phenomenon that has been explained by incomplete lineage sorting or ancient hybridization [21, 22]. European, Siberian, and American *B. priscus* mitogenomes were shown to be phylogenetically closer to *B. bison* than to *B. bonasus* [7, 14, 23].

Ancient DNA studies have the potential to better resolve taxonomy than paleontological studies, in particular at the species level, and have revealed a far more dynamic picture of megafaunal communities, biogeography, and ecology, including repeated localized extinctions, migrations, and replacements (e.g., [12] and citations therein). To better understand the phylogeography and evolution of the wisent and to reconstruct its origin, we performed a study of the mitochondrial genome over the past 50,000 years (50 kyr) across Europe and Asia. Using Pleistocene skeletal remains from Siberia (Yakutia), the Caucasus and Western Europe (France, Switzerland), as well as Neolithic and Medieval samples from France and pre-bottleneck *B. bonasus* samples from the early 20th century from the Polish lowland and the Caucasian highland lines, we constructed a phylogenetic framework based on both the hypervariable region (HVR) of the mitochondrial DNA as well as complete mitochondrial genomes of selected specimens. Our results give new insights into the climate-driven dynamics of the bison in Europe.

## Results

### The evolution of the mitochondrial HVR

**Fig. 1:**
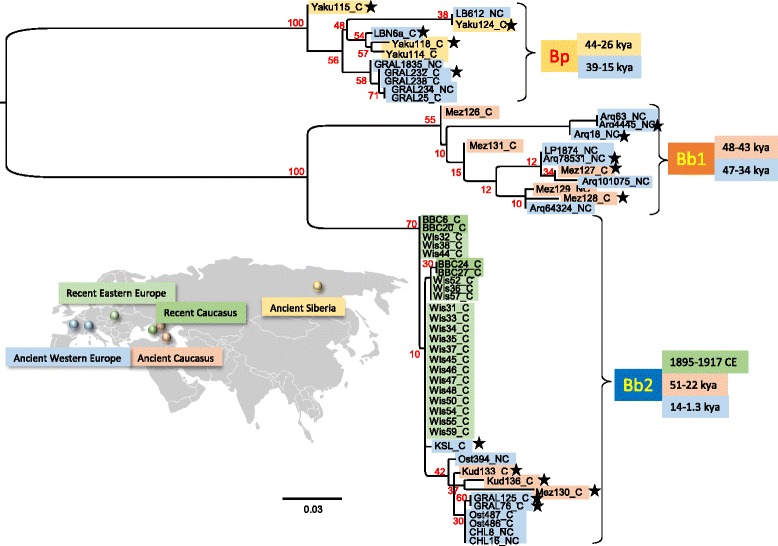
Maximum likelihood phylogeny of the ancient Bison hypervariable region. Maximum likelihood analyses of the hypervariable region produced in this study using PHYML, a HKY + I + G substitution model and 500 bootstraps. The bootstrap support of the nodes is indicated in red. The geolocalization of the analyzed samples is represented using a color code to distinguish five origins and time periods as represented on the Eurasiatic continent map (see Additional file 6: Figure S4 for a map of the distribution of the Western European sites). Three clades can be clearly distinguished, the *Bison priscus* (*Bp*) clade and two *Bison bonasus* clades (Bb1 and Bb2). The scale corresponds to the number of nucleotide substitutions per site. The samples that have allowed amplification of all four PCR fragments as represented in Additional file 2: Figure S1 are indicated by the suffix _C whereas those for which one or more fragments were missing are indicated by the suffix _NC. The stars indicate the samples that were used for the full mitogenome analysis presented in Fig. 3

We analyzed 66 paleontological and 25 historical specimens (Additional file 1: Table S1) targeting the mitochondrial HVRs with four fragments of a maximum of 150 bp. We obtained a 367 bp-long sequence from 43 specimens, and smaller sequences from 13 additional ones (Additional file 2: Figure S1). A maximum likelihood phylogenetic tree was constructed using these sequences (Fig. 1). Three clades can be clearly distinguished. The first clade *Bp*, which is more divergent from the other two, comprises samples from Pleistocene Yakutia (Siberia) and from southern France (La Berbie and lower stratigraphic layers of Igue du Gral), dating from 44 to 26 kya and from 39 to 15 kya, respectively. Clade *Bp* corresponds to the *B. priscus* lineage previously described for Siberia, North America, and Europe [7, 23]. The French and Siberian sequences of this clade are phylogenetically close and lack a phylogeographic structure. This reveals that a relatively homogeneous population of steppe bison was distributed during the Late Pleistocene not only in Siberia and northern America, but also throughout the entire northern part of the Eurasian continent up to its most-western part, France.

The two other clades, *Bb1* and *Bb2*, are more closely related to the extant wisent *B. bonasus*. The *Bb1* clade, significantly divergent from *Bb2*, contains most of the specimens from the Mezmaiskaya cave northwest of the Caucasus in Russia, dating from ca. 48–43 kya, as well as seven specimens from southern France (Arquet, Les Plumettes), dating from ca. 47–34 kya. Thus, the geographical range of the *Bb1* clade stretched from at least the northern Caucasus to the south of France, from 48 to 34 kya during the overall mild Marine Isotope Stage (MIS) 3 (ranging from 57 to 29 kya [24], http://www.lorraine-lisiecki.com/LR04_MISboundaries.txt). None of the haplotypes of the *Bb1* clade was found in the more recent specimens, suggesting that the corresponding population may have been the first to disappear. This *Bb1* population in France was apparently replaced by the steppe bison of the *Bp* clade around 30 kya, the latter remaining there throughout the glacial period of MIS2 (ranging from 29 to 14 kya [24], http://www.lorraine-lisiecki.com/LR04_MISboundaries.txt).

Clade *Bb2* includes both ancient specimens from the Caucasus and Western Europe as well as all recent and extant *B. bonasus* specimens. Nearly all ancient samples belonging to this clade are distinct from the more recent populations and include a ca. 49 kyr-old specimen from the Mezmaiskaya cave, two specimens from the Kudaro cave in the central part of the southern slope of the Greater Caucasus dated at 38 and 22 kya, and specimens from Western Europe – one from the Kesslerloch cave (Switzerland) dated at 14 kya, and, in France, two bison from the upper stratigraphic sequence of the Gral dated at 12 kya, two 5.2-kyr-old bison samples from the Neolithic site of Chalain, as well as three medieval (7th to 8th century CE) specimens from Alsace. The members of this clade represent the western European Pleistocene-Holocene lineage of *B. bonasus* and display a high mitochondrial diversity. This lineage appears to have replaced the *B. priscus* lineage, at least in France, at the end of the Upper Pleistocene between 15 and 12 kya, coinciding with the onset of a more temperate climate, and to have persisted in France up to the Middle Ages. Apart from the sequence found in the sample from Kesslerloch (12.2 kya), none of the ancient Bb2 sequences are present in the extant mitochondrial gene pool.

Within the *Bb2* clade, a compact group of closely related sequences, are the Upper Pleistocene Kesslerloch specimen and the 1898–1917 pre-bottleneck wisents from both Poland and the Caucasus that almost became extinct at the end of the First World War. Five out of 24 of these pre-bottleneck bison have a HVR sequence identical to that of extant *B. bonasus*, whereas the rest differ from the extant sequence by only one or two single nucleotide polymorphisms (SNPs). Thus, the pre-bottleneck mitochondrial diversity appears only slightly higher than at present and much lower than that observed in older samples. The 14-kyr-old Kesslerloch specimen reveals the first occurrence of the mitogenome lineage of the extant *B. bonasus* population. Thus, the modern population corresponds to a minor fraction of the diversity that was present in Europe during the Late Pleistocene when *B. bonasus* replaced *B. priscus.*

A major reduction in the intrapopulation diversity is apparent from the Late Pleistocene to the early 20th century (Additional file 1: Table S2). The Pleistocene populations of Siberia, the Caucasus, and Europe are characterized by a high diversity at both the haplotype (*H* = 1.00) and nucleotide levels (as estimated with Pi and various Theta estimators), the nucleotide diversity of the European *B. priscus* being lower than that of its Siberian population. The Western European Holocene population of *B. bonasus* experienced a major reduction of its diversity between the Middle Ages and the beginning of the 20th century.

**Fig. 2:**
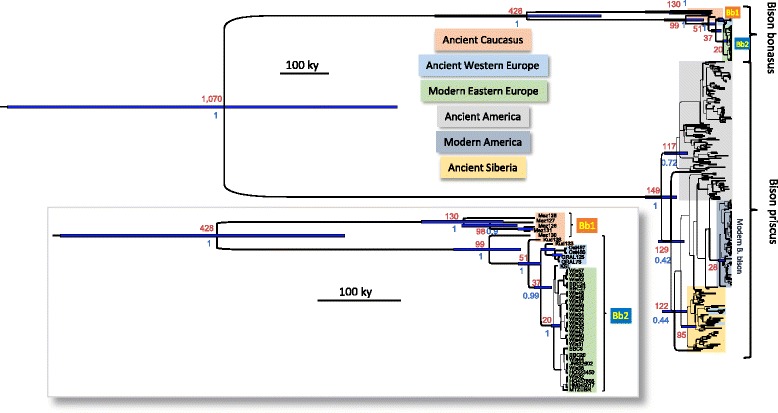
Bayesian phylogeny of Bison hypervariable region. All dated and complete ancient Bison sequences produced here and in a previous *B. priscus* analysis [7] were aligned and reduced to the 367 bp sequence targeted herein alongside modern *B. bonasus* and *B. bison* sequences. A Bayesian phylogenetic analysis was performed using Beast to estimate the age of the nodes from temporally spaced sequence data. The age of the nodes (in kya) is indicated in red, whereas the blue bars represent the 95 % highest posterior density interval of these ages. The color code representing the origin of the various samples is as indicated. The inset on the lower part of the figure represents a magnified view of the *B. bonasus* branches of the tree. The posterior probability of the nodes is indicated in blue and the thickness of the branches is proportional to this posterior probability

We performed a phylogenetic analysis of the HVR under the Bayesian framework estimating mutation and population history parameters from temporally spaced sequence data using all dated ancient and modern bison HVR sequences present in GenBank in 2015 (Additional file 1: Table S3) in combination with the data from the current study (Fig. 2). The resulting tree shows a bifurcation between the *Bison bonasus* and *Bison priscus/bison* mitogenome lineages about 1.0 million years ago (mya) (95 % highest posterior density interval (HPD): 1.5–0.7) (Table 1 and Fig. 2). The most recent common ancestor (MRCA) of the Eurasiatic and the American *B. priscus* clades is estimated here at 151 kya (193–119), which is in agreement with a previous estimate of 136 kya (164–111) that was also based on the HVR [7]. *B. priscus* from America and Siberia group in distinct clades, and the modern American bison descended from a small subgroup of a once diverse American population, as previously proposed [7]. The Late Pleistocene European *B. priscus* corresponds to a subset of the Siberian population.

**Table 1:** Node age estimates and clock rate estimates obtained through Bayesian analyses

Age estimates of the nodes (kya)		
Node	Age from HVR (95 % HPD)	Age from mitogenome (95 % HPD)
Root Bovina	1080 (1550–700)	927 (1064–790)
*Bos Taurus*/*B. bonasus*	–	768 (886–657)
*B. indicus*/*B. taurus*	–	157 (185–130)
*B. grunniens*/*B. priscus*	–	317 (370–265)
*B. priscus*	151 (193–119)	114 (134–95)
*B. bison*	21 (16–27)	11 (7–14)
All *B. bonasus*	438 (643–284)	246 (283–212)
*B. bonasus* Bb2	105 (158–75)	87 (93–71)
Clock rate estimates (per site * year)		
Partition	Rate from HVR (95 % HPD)	Rate from mitogenome (95 % HPD)
HVR	5.4 (3.9–6.9) × 10^–7^	4.6 (3.7–5.6) × 10^–7^
RNA	–	2.5 (2.1–3.0) × 10^–8^
Coding (first to second positions)	–	2.4 (2.0–2.8) × 10^–8^
Coding (third position)	–	9.9 (8.5–11.4) × 10^–8^

*kya* thousand years ago, *HPD* highest posterior density interval, *HVR* hypervariable region

The phylogenetic analyses of the *B. bonasus* haplotypes reveal that the ancient Caucasian population had a deep root and was highly diverse. Based on the HVR, the age of the MRCA of the *Bb1* and *Bb2* clades is estimated at 438 (643–284) kya, which is significantly older than the MRCA of *B. priscus* (Fig. 2 and Table 1). This observation indicates that the Upper Pleistocene Caucasian population had a deeper root than the contemporaneous and more northern *B. priscus* population, even though the latter may have occupied in the past a much larger territory from Western Europe to the American continent. The *Bb2* clade encompasses several branching events, the separation of a branch represented by a ca. 50-kyr-old northern Caucasus specimen from Mezmaiskaya occurring first, i.e., 105 (158–75) kya, followed, 61 (89–43) kya, by the separation of a branch represented by a ca. 37.8-kyr-old specimen from the Kudaro cave in the southern Caucasus. Then, 42 (60–26) kya, the subgroup comprising a 22.2-kyr-old specimen from the Kudaro cave as well as all French specimens between 12.4 and ca. 1.2 kya separated from the haplogroup encompassing the modern *B. bonasus* sequences. Finally, in this latter haplogroup, the 14-kyr-old Kesslerloch specimen and the early 20th century Central European and Caucasus specimens have a MRCA estimated at 16 (33–14) kya. This sequential order of the radiation events and the previously mentioned reduction of the population diversity indicates that, at the transition between the Pleistocene and the Holocene, Western and Central Europe were populated by a subset of a wisent population established in an area of Southwest Asia including the Caucasus, and that only a part of this population survived into the 20th century.

### The evolution of the Bison clades based on entire mitogenomes

**Fig. 3:**
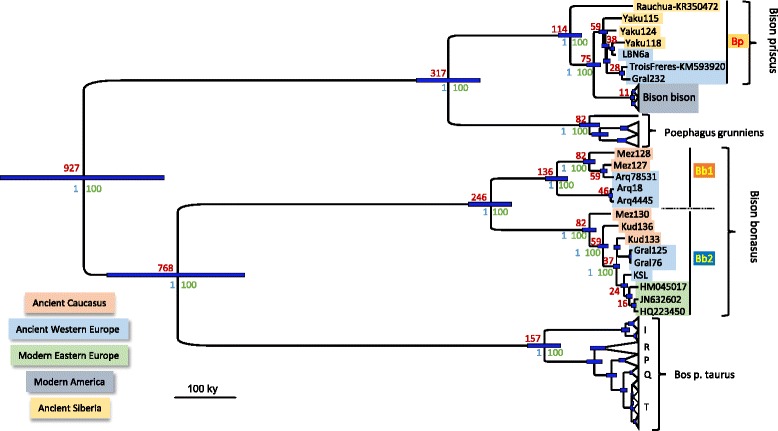
Bayesian phylogeny of complete mitogenomes of *Bos* and *Bison*. We used the complete mitogenomes of ancient *Bison* obtained herein as well as the two published *B. priscus* mitogenomes, and all modern *Bison bison*, *Bison bonasus*, *Poephagus grunniens*, and *Bos primigenius taurus* mitogenomes available in GenBank in 2015 totaling 420 sequences. The *B. bison*, *P. grunniens*, and *B. p. taurus* sequences have been collapsed to preserve only their subclade structure. The estimate of the age of the nodes, in kya, are indicated in red, with the 95 % highest posterior density interval indicated by blue bars. The statistical supports of the nodes are indicated in blue (Bayesian posterior probability) and in green (bootstrap values of a maximum likelihood phylogeny performed using RaXML)

To render this phylogeny and dating scenario more robust, we used biotinylated RNA probes synthesized from the complete mitogenome of *B. p. taurus*, and optimized a sequence capture approach to recover complete mitogenome sequences from 16 well-preserved 50- to 12-kyr-old specimens representative of the various clades and radiation events defined above (see coverage information in Additional file 1: Table S4). We performed a Bayesian phylogenetic analysis of these mitogenomes and of two recently published *B. priscus* mitogenomes [14, 23] together with those of modern *B. bison* and *B. bonasus*, *B. p. taurus*, and *P. grunniens* complete mitogenomes present in GenBank in 2015 (Additional file 1: Table S4 and Fig. 3). As previously observed with modern sequences [9, 21, 22], the *B. bonasus* mitogenome lineage is more closely related to the *Bos p. taurus* lineage than to the *B. priscus–B. bison* lineages. The Bayesian analysis reveals, however, that there is a significant overlap (35–40 %) of the 95 % HPD intervals of the dates estimated for the node separating the *Bos p. taurus–B. bonasus* and the *B. priscus–B. bison* lineages, estimated here at 927 (1064–790) kya, and the node separating the *B. p. taurus* and *B. bonasus* lineages, estimated here at 768 (886–657) kya. Such an overlap suggests that the two bifurcation events may have occurred within a relatively short evolutionary period, thus increasing the likelihood that these two events preceded the major separation of the *B. p. taurus* and *Bison* species. This peculiar affiliation pattern of mitogenomes renders the incomplete lineage sorting hypothesis a parsimonious interpretation (Additional file 3: Figure S2). The *Bison priscus/bison* and yak (*P. grunniens*) mitogenome lineages separated from a common ancestor dated at ca. 317 (370–265) kya. For the *Bison* mitogenomes, the dates of the nodes estimated with complete mitogenomes are often younger than those estimated using only the HVR (Table 1). For instance, the common ancestor of the *Bb1* and *Bb2* clades is estimated at 246 (283–212) kya when comparing full mitochondrial genomes, rather than 438 (643–284) kya calculated from only HVR sequences. Similarly, the common ancestor of the Eurasiatic *B. priscus* and the modern American bison is estimated at 114 (134–95) kya rather than 151 (193–119) kya. In contrast, the various branching events within the *Bb2* clade show non-significant differences (given the overlap of the HPD) between the two series of date estimations: 82 (93–71) kya instead of 105 (158–75) kya for the ancestor of the North and South Caucasian specimens, 59 (68–51) kya instead of 61 (89–43) kya for the ancestor of the South Caucasus and Holocene European specimens, and 35 (42–31) kya instead of 42 (60–26) kya for the ancestor of the Holocene European specimens. The discrepancies between the date estimates for several nodes depending on the genetic region used may be partly due to the differences in the number of individual sequences studied in the two series. The major source of discrepancy, however, appears to be due to an irregular rate of evolution of the HVR. Indeed, even though the clock rates estimated for the HVR are similar when either the HVR alone or as a partition of the complete mitogenome is used (5.4 (3.9–6.9) × 10^–7^ and 4.6 (3.7–5.6) × 10^–7^ per site per year, respectively), the individual HVRs show distinct evolutionary rates. When the distribution throughout the mitogenome of the SNPs distinguishing individual bison mitogenomes from the *B. p. taurus* mitogenome as an outgroup are compared, the number of SNPs accumulated in the HVR can vary up to two-fold between individual sequences, whereas the rest of the mitogenome is equally distant to the outgroup (Additional file 4: Figure S3). For example, there are twice as many mutations accumulated in the HVR subregion (15900–16100) of the Mez 128, Gral 232, and Yaku 118 mitogenomes than in the ones of the Mez130 and Gral 125 specimens. Whatever the underlying mechanism responsible for these differences of the evolutionary rate of this particular region of the mitogenome, this phenomenon limits the reliability of the dating estimations based solely on the HVR.

Complete mitogenomes confirm the observation made with the HVR sequences that the European *B. priscus* specimens from the Late Pleistocene were more homogeneous genetically than the contemporaneous Siberian population. Indeed, the two ca. 14–19 kya French mitogenomes from the Gral (Gral232), and Trois-Frères cave [23] are very similar, with only 19 SNPs distinguishing them, whereas the Siberian mitogenomes (all three Yaku and Rauchua [14]) are much more divergent, with 8-fold more SNPs distinguishing them (ca. 150 SNPs separating Rauchua from the Yaku and ca. 50 SNPs separating the Yaku samples from each other). The more ancient, ca. 38 kya French sample of La Berbie (LBN6a) is, however, more distant from the two French *B. priscus* (ca. 50 SNPs) and more closely related to one of the Siberian samples (Yaku118, 23 SNPs). Strikingly, the age of the MRCA of the three Yakutian and three French *B. priscus* samples is estimated at 59 (66–52) kya. Since the MRCA of these sequences is also the MRCA of the group of HVR sequences comprising all other French *B. priscus* samples (Fig. 1), this indicates that the steppe bison inhabiting France between 39 and 14 kya originated from a migration from North-East Eurasia that occurred not earlier than 59 (66–52) kya. Presumably, the low genetic diversity of the Western European *B. priscus* population is due to a founder effect during this migration.

The phylogenetic relationships between the *B. bonasus* mitogenomes indicate that modern wisent in central Europe are more closely related to the Late Pleistocene/Holocene Western European population than to the Late Pleistocene Caucasian population. Indeed, the 14-kyr-old specimen from the site of Kesslerloch at the Swiss-German border is closely related to modern wisent, with only 15 SNPs distinguishing the ancient and modern mitogenome sequences. This demonstrates that the range of the ancestral population of the modern wisent encompassed Middle Europe 14 kya. The Late Pleistocene Caucasian population was highly diverse with a deep root separating most of the specimens from the Mezmaiskaya cave in the northern Caucasus, belonging to the *Bb1* clade, from the specimens from the Kudaro cave in the southern Caucasus, belonging to the *Bb2* clade and including a specimen from the northern Caucasus. About 300 SNPs distinguished the Bb1 (Mez127, Mez128) and the Bb2 (Mez130, Kud133, Kud136) mitogenomes and approximately 100 SNPs distinguished the northern (Mez 130) and southern Caucasian (Kud133, Kud136) mitogenomes of the *Bb2* clade. In contrast, the members of the *Bb2* clade in Western Europe were more similar, with only 39 SNPs distinguishing the French (Gral125) and Swiss (KSL) specimens and three SNPs distinguishing the two French samples (Gral76, Gral125), in agreement with the reduction of diversity observed when comparing the HVR of the Western European and Caucasian *B. bonasus* populations. Thus, complete mitogenomes confirm the observations from the HVR analyses of a larger sample size while providing a more accurate phylogenetic analysis, in particular with respect to the dating of the MRCA of the various mitogenomes.

## Discussion

### European bison population turnover during the late Pleistocene and the Holocene

**Fig. 4:**
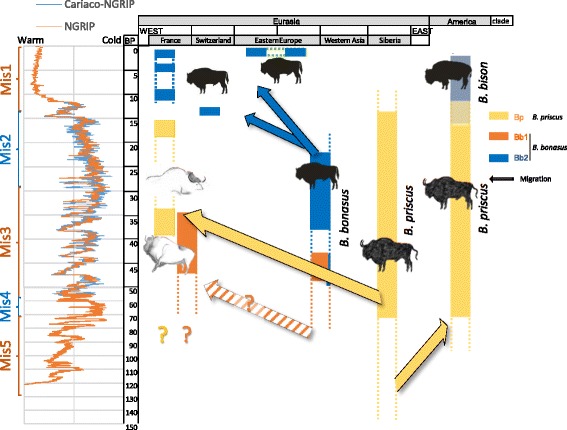
Schematic representation of the distribution through time and space of the various mitogenome clades. The geographic regions are represented on the abscissa and the time scale on the ordinate. The ascertained presence of the various mitochondrial haplogroups are represented by solid boxes, whereas the dotted lines indicate possible temporal extension of the presence of these clades. The left side shows the climatic fluctuations as inferred from the North Greenland Ice Core Project (NGRIP) [25] and the combined Caribbean Cariaco basin and NGRIP data as shown in [12], as well as the Marine Isotope Stage (MIS) as defined by Lisiecki and Raymo [24] (http://www.lorraine-lisiecki.com/LR04_MISboundaries.txt). The proposed migrations are indicated by solid arrows. The hatched arrow indicates a possible migration of the *Bb1* clade that populated Western Europe from a southern refugee before the time period analyzed herein. The genetic identity of the bison that, according to the fossil record, populated Western Europe prior to 60 kya is not known, but climatic fluctuations may have triggered additional expansions and contractions of different populations of *B. priscus* and *B. bonasus*. (Drawings: E-M Geigl)

Our results enable us to propose a scenario for the evolutionary history of the bison in Europe that is related to the climatic fluctuations and the resulting environmental changes of the Late Pleistocene and the Pleistocene/Holocene transition as summarized in Fig. 4. We observe striking regional and temporal differences in the major clades and distinguish three periods, particularly in France. The first period, from at least 47 kya to about 34 kya, was characterized by the dominance of a divergent *B. bonasus* lineage belonging to the *Bb1* clade in both southern France (Arquet and Plumettes, seven *Bb1* out of seven samples) and the northern Caucasus (Mezmaiskaya, five *Bb1* and one *Bb2* out of six samples). This lineage was absent from the samples from later periods, indicating that the corresponding population was the first to disappear. For the same time period, the steppe bison mitotype *Bp* was the sole mitotype found in Siberia and northern Eurasia (27/27 samples dated from 66 to 34 kya [7]). This period, encompassing most of MIS3, is characterized by oscillating shorter glacial and longer interstadial periods, the latter lasting more than 1000 years (glacial interstadial GI15 to GI8 [12, 25] and Fig. 4). During the warmer periods, southern France and the northern Mediterranean coast were covered by non-continuous deciduous coniferous forests, and central France and central Europe by coniferous open woodland [26]. The steppe bison *B. priscus* was dominant in France during the following period, which lasted until the end of the Last Glacial Maximum (LGM) at 14.7 kya (7/7 samples, plus 1/1 in [23]). These bison were closely related to the Siberian sample from Yakutia from the same period, the whole mitogenome of which we analyzed (for example, the Yaku118 mitogenome is 99.9 % identical to that of LBN6a and 99.4 % identical to that of Gral232). HVR comparisons between the French and Siberian and North American samples [7] reveal that the French *B. priscus* mitotypes are closely related to those of the Siberian samples (Fig. 2), indicating that the western European territory was colonized by a subpopulation of the steppe bison from the northern Eurasian continent. The age estimated from full mitogenomes for the MRCA of the French and Siberian samples is 59 (66–52) kya, indicating that this colonization involved a population that separated from the Siberian population more recently than ca. 59 kya and suggesting a lower limit for the arrival in France of these steppe bison. In contrast, specimens older than 70 kya assigned to *B. priscus* in the fossil record of Western Europe presumably must have belonged to a distinct population that was not the direct ancestor of this *B. priscus* population that occupied Western Europe during the cold spells of MIS2. At the end of MIS3, around 32 kya, the climate became colder on average and the warmer interstadials were shorter, lasting only a few hundred years. Then, between 27 and 14.7 kya, a second, long glacial period followed that comprised two phases. In the first phase, the tree cover was patchy and incomplete, with a high proportion of steppe vegetation, whereas the second, a full glacial phase, was characterized by sparse grassland and open steppe tundra in southern and northern Europe, respectively [27]. While during this period the steppe bison *B. priscus* occupied the territory previously occupied by *B. bonasus* in Western Europe, *B. bonasus* remained nevertheless present in the southern Caucasus, even during the LGM. Indeed, in our samples, two out of two specimens from the southern Caucasus belonged to the *Bb2* clade, which we found to be present at low frequency at an earlier period in the northern Caucasus. Finally, during the third period, starting at the end of the MIS2 and lasting up to the present, *B. bonasus* of the *Bb2* clade expanded again into Western Europe, as we detected it in the 14-kyr-old specimen from Switzerland at the beginning of the Bølling-Allerød interstadial period (14.7–12.7 kya). The more recent specimens from France belonged, without exception, to the *Bb2* clade (7/7, dated between 12 kya and the Middle Ages). Strikingly, the sedimentary sequence of the French site Igue du Gral recorded a population replacement: all specimens older than ca. 15 kya, before the Bølling-Allerød interstadial, belong to the *B. priscus Bp* mitotype (5/5), whereas all more recent ones, coinciding with the onset of a more temperate climate at the end of the last glacial event (end of MIS2), belong to the *B. bonasus Bb2* mitotype (2/2, *P*_val_ < 0.05 with Fisher’s test). The ancient French *Bb2* population is significantly different from the one comprising the modern wisent and the ancient Kesslerloch specimen (Figs. 1–3). Moreover, this subclade was not detected later than the Middle Ages, suggesting that it went extinct in Western Europe with the disappearing local wisent. In contrast, the distinct *Bb2* mitotype of the 14-kyr-old sample from Kesslerloch continued to exist up to present time. It is the only remaining mitotype detected in both present-day wisent as well as in the specimens from Poland and the Caucasus from the beginning of the 20th century prior to the last major bottleneck of the First World War.

The marked climatic variations that occurred during the Pleistocene implied drastic environmental changes that triggered the profound reorganization of floral and faunal biomes at different geographic and temporal scales. Response of biota to climatic stimuli was regionally/continentally distinct, sometimes occurring synchronously but most often diachronously (e.g., [2, 28]). The repeated cyclical climate changes progressively promoted landscape renewal, locally modifying, obliterating, and creating specific and varied ecological niches [28]. The various expansions and contractions of bison populations in response to these climatic fluctuations led to apparent alternating occupations of Western Europe by either *B. priscus* or *B. bonasus* during the last 50 kyr, each presumably respectively originating from either a northeastern territory including Siberia, or a southern territory including the Caucasus. We hypothesize that these alternating occurrences result from climate-driven changes of two different habitats to which *B. priscus*, a grazer, and *B. bonasus*, a mixed feeder, were adapted, i.e., the open tundra-steppe for the first and open woodlands for the second. Indeed, the diet of *B. priscus* during the LGM included typical steppe and grassland (C_3_) vegetation and lichens [29], whereas the wisent’s diet in the Holocene was more flexible and included a higher content of shrubs [18]. Similarly, on the American continent, *B. priscus* adapted to the climatic and environmental changes of the Holocene and evolved in two recently divergent forms, the plain bison thriving on the grasslands of the Great Plains and the wood bison inhabiting the boreal forest in North America. This suggests that, on the Eurasian continent, the competition with *B. bonasus*, a species seemingly better adapted to a more temperate environment, may have prevented a similar adaptation of *B. priscus* to habitat changes during the warmer periods. Finally, in Western Europe, local variations in ecological and physical barriers could have affected the speed of bison population turnover as a reaction to climatic shifts. In the future, a higher resolution genetic study involving a much higher sample number with denser time and space sampling may provide a more accurate and nuanced view of these population turnovers.

The mitochondrial lineages of *B. bonasus* that were present 40 kya have an older root than those of *B. priscus* (246 (283–212) vs. 114 (134–95) kya). This indicates that the *B. bonasus* did not experience as severe a bottleneck prior to 100 kya as the population reduction the steppe Bison experienced between 150 and 100 kya, before later thriving in northern Eurasia, Beringia, and in North America between 80 and 20 kya. All the various population expansions and contractions that occurred in response to the climatic and environmental changes characterizing the late Pleistocene and the transition to the Holocene gave rise to a reduction of the population diversity and to the extinction of lineages, like the *Bb1* lineages, which apparently was the first to disappear, and the *Bp* lineage, which disappeared at the beginning of the Holocene on the Eurasian continent. Finally, the reduction of the diversity of the *Bb2* lineage seems to involve both migrations and local extinctions with the major and most severe reductions occurring between the Early Holocene and historic times, most likely owing to human impact through hunting pressure and habitat fragmentation.

**Fig. 5:**
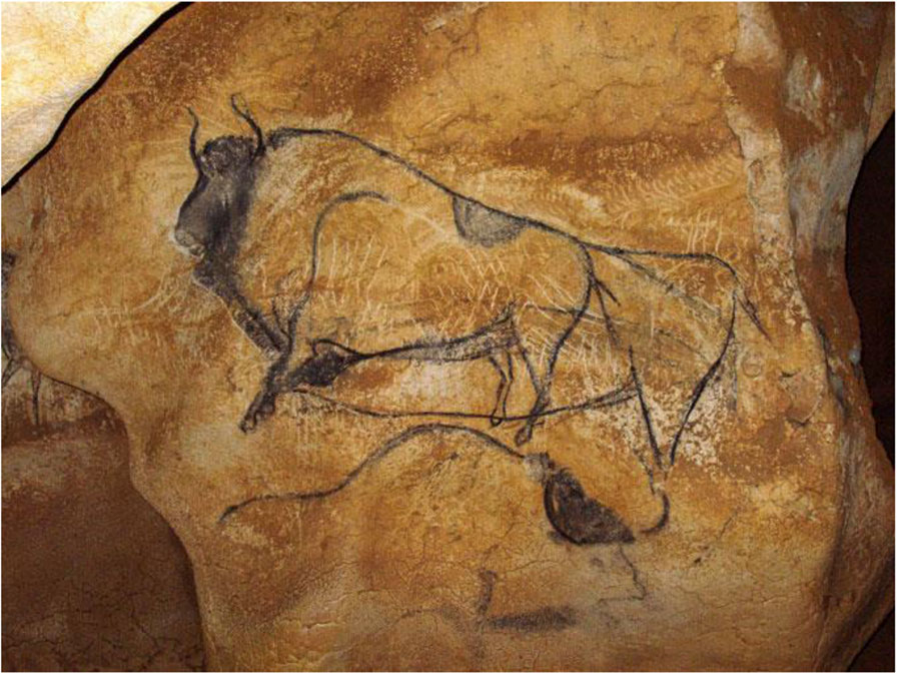
Prehistoric painting of bison in the cave of Chauvet-Pont d’Arc, Ardèche, France. The paintings are the so-called “Bison of the pillar” in the “End Chamber” of the Chauvet cave. The charcoal of both paintings have been radiocarbon dated at 38.5–34.1 kya for the upper bison, and at 36.3–34.6 kya for the lower bison [30]. We consider, based on criteria stated by Spassov [19], that the “great bison” in the upper part represents *B. bonasus* with a highly positioned head, curved horns, a moderately large hump and a weak mane, and rather equilibrated body proportions between the front and the rear. The lower part would represent *B. priscus* with its large hump, its low head position, its abundant mane, and crescent-shaped horns; although somewhat faded in the image, the steep incline of the back-line and stronger hindquarters can be made out. Printed with permission of the Centre National de Préhistoire, France. (Copyright: French Ministry of Culture and Communication, archeologie.culture.fr/chauvet; Arnaud Frich, Centre National de Préhistoire/MCC).

Numerous cave art representation in southern France and northern Spain, and also in the Caucasus suggest realistic depictions of both the steppe bison and the wisent [19]. We consider likely that the paintings of bison, the so-called “bison of the pillar”, in the cave Chauvet-Pont d’Arc in France depict the two types of bison distinguished by the shape of horns and back lines (Fig. 5). The upper image on the pillar, dated at 38.5–34.1 kya [30], could represent a wisent, and the lower image, dated at 36.2–34.6 kya [30], a steppe bison. These dates coincide with the period (39–34 kya) in which the two bison forms overlap in our dataset from southern France, in the vicinity of these paintings. Within this time frame, wisent population would have been in decline and steppe bison population would have been expanding.

### Radiation of the *Bos* and *Bison* lineages

Since the initial classification of Linnaeus in 1758 of *Bos* and *Bison* within a single genus, there has been debate about whether they should be placed in separate genera [31]. Indeed, the members of these genera can still be crossed, despite reduced fertility in some combinations of crosses, and *B. p. taurus* genetic material can be found in a number of present-day American bison and wisent [32, 33]. Thus, gene flow could have occurred between the various lineages, in particular in the early phases of their differentiation. Such gene flow between separated populations has already been considered as an alternative possibility to incomplete lineage sorting to explain that the mitogenome of *B. p. taurus* was closer to that of *B. bonasus* than to that of *B. bison* [21, 22]. The phylogenetic estimates of the age of the various common ancestors of these two lineages indicate, however, that the most parsimonious interpretation of incomplete lineage sorting suffices to account for the relative affiliations of these mitogenomes (Additional file 3: Figure S2). Indeed, as mentioned above, the age estimates indicate that the MRCA of the *Bos* and *Bison* mitogenome lineages is not much older than that of the *B. p. taurus* and *B. bonasus* lineages, which are 927 (1064–790) kya and 768 (886–657) kya, respectively, with a 35–40 % overlap of the confidence interval (95 % HPD). This indicates that the two bifurcation events have occurred within a relatively short evolutionary period. Rapid speciation during this short evolutionary period appears unlikely since these species have still not yet totally lost interfertility almost a million years later. Our radiation date estimates are within the same range of the earliest fossils that were clearly attributed to the *Bos* genus and that are dated at 1 mya [34]. They are also consistent with the analysis of the complete genome of a modern wisent that estimated that the wisent and bovine species diverged between 1.7 and 0.85 mya through a speciation process involving an extended period of limited gene flow with some more recent secondary contacts posterior to 150 kya [35]. Thus, incomplete lineage sorting of mitogenomes in a metapopulation of the *Bos* and *Bison* ancestors during the period of divergence of these species could account for the affiliation patterns of these mitogenomes without the need to postulate a more recent post-speciation gene flow. It is interesting to note that this radiation event could be coincident with the onset and intensification of high-latitude glacial cycles (100 kyr-periodicity) around 1.2–0.8 mya. Incomplete lineage sorting, however, does not preclude later sporadic introgression of nuclear DNA at any point up to the present owing to the persistent interfertility between these species, as evidenced by the detection in the wisent genome of ancient gene flow from *B. p. taurus* [35, 36].

## Conclusion

The analysis of DNA preserved in ancient bison remains from Eurasia covering the last 50,000 years allowed us to retrace some of the population dynamics that took place during the Late Pleistocene and the Holocene, including population migrations, extinctions, and replacements. We could trace back the origin of the wisent to Eastern Europe, including the Caucasus. We observed several alternating expansion waves of wisent and steppe bison in Western Europe that were related with climatic fluctuations. The wisent was prevalent when the climate was more temperate leading to a more forested vegetation, similar to present-day Western Europe, which is compatible with its reintroduction in this area. In contrast, the steppe bison population originating from northern Eurasia was predominant in Western Europe during the colder periods of the Late Pleistocene with their open environments. These fluctuations may have been recorded in Paleolithic cave paintings, in particular in the cave of Chauvet that had been occupied by humans over a long period and where two distinct bison types are depicted.

## Methods

Details of samples are described in Additional file 1: Table S1, Additional file 5: Document S1 and Additional file 6: Figure S4. All pre-PCR procedures were carried out in the high containment facility of the Jacques Monod Institute physically separated from areas where modern samples are analyzed and dedicated exclusively to ancient DNA analysis using the strict procedures for contamination prevention previously described [37].

### DNA extraction

The external surface of the specimens was removed with a sterile blade to minimize environmental contamination. For each bone sample, roughly 0.2 g was ground to a fine powder in a freezer mill (Freezer Mill 6750, Spex Certiprep, Metuchen, NJ), which was then suspended in 2 mL of extraction buffer containing 0.5 M EDTA, 0.25 M di-sodium hydrogen phosphate (Na_2_HPO_4_) at pH 8.0, 0.14 M 2-mercaptoethanol, and 0.25 mg/mL of proteinase K and incubated under agitation at 37 °C for 48 hours. Blank extractions were carried out for each of eight total extraction series.

Samples were then centrifuged and the supernatant was purified with the Qiaquick PCR Purification Kit (Qiagen, Hilden, Germany), as previously described [37].

### PCR amplification of the HVR

Primers were designed to target conserved regions with minimal primer dimer propensity from a multiple alignment of all the bison mitochondrial sequences present in GenBank in 2012 using the software Oligo 7 as previously described [38, 39], and were then tested for efficiency and dimer formation using quantitative real-time PCR (qPCR) [38, 39]. Four primer pairs amplifying short (118–152 bp) subregions of the mitochondrial HVR were selected (Additional file 1: Table S6 and Additional file 2: Figure S1). To protect against cross-contamination between samples, we used the UNG-coupled quantitative real-time PCR system [40] and, to avoid the production of erroneous data due to the presence of bovine DNA in reagents, reagent decontamination was performed as previously described [41]. Amplifications were performed in a final volume of 10 μL containing 2 mM MgCl_2_, 1 μM primers, 0.04 mM of dA/G/CTPs, 0.08 mM of dUTP, 0.01 U/μL of UNG, 1 U/μL of FastStart Taq (Roche Applied Science, Penzberg, Germany), and 1× qPCR home-made reaction buffer [41]. Blank amplification controls were included for each amplification. In total, 415 amplification blanks were carried out during the various amplifications of 85 samples. No products were observed in any of the amplification blanks. Amplifications were performed using a LightCycler 1.5 (Roche Applied Science) with the following cycling program: 15 min at 37 °C (carry-over contamination prevention through digestion by UNG of dUTP-containing amplicons), 10 min at 95 °C (inactivation of UNG and activation of the Fast Start DNA polymerase) followed by 60 cycles at 95 °C for 15 sec, at 60 °C for 40 sec (for primer pairs Bon1, 2 and 3), and 95 °C for 15 sec, or 56 °C for 15 sec and 67 °C for 20 sec (for primer pair BB3r4m), followed by a final melting curve analysis step. All extracts were tested for inhibition as previously described [42]. Each sequence was determined on both strands from at least two independent amplifications using capillary electrophoresis sequencing. Sequencing data analyses were performed using the software Geneious 6.1.8 [43].

### Capture and sequencing of whole mitogenomes

#### Library preparation

Dual barcoded libraries for Illumina sequencing were constructed in the dedicated ancient DNA facility at the Jacques Monod Institute (Paris, France) using a double-stranded procedure previously described [37, 44]. Ancient DNA extracts were quantified on a Qubit 2.0 Fluorimeter (Thermo Fisher Scientific, Waltham, MA). Libraries were prepared using between 3 and 124 ng total DNA. The USER Enzyme, End-repair and adapter ligation mixtures were decontaminated using Ethidium monoazide [45] to inactivate bovine DNA associated with BSA present in these reagents that could interfere with the final results. Prior to library preparation, uracil residues were removed through treatment with 1.5 units/30 μL reaction volume of USER Enzyme (New England Biolabs, Ipswich, MA) for 60 min at 37 °C in NEBNext End Repair buffer (New England Biolabs). End repair was performed using the NEBNext End Repair Module, using only half of the enzyme mix recommended by the manufacturer. End-repaired DNA was purified on MinElute columns using the Qiagen Gel extraction protocol, and DNA was eluted twice, each with 16 μL of γ-irradiated H_2_O [41]. Blunt-end ligations were performed using the NEBNext Quick Ligation Module (New England Biolabs); 1 μL each of N700 and N500 barcoded adaptors (20 μM) from the Nextera XT series (Illumina Inc., San Diego, CA) and 1.5 μL of T4 ligase were added to the purified reactions and incubated 30 min at 20 °C in ligation buffer. After ligation, elongation of the adapters was performed by adding 1 volume of OneTaq DNA polymerase 2× Master Mix (New England BiolabsI) and incubating for 20 min at 60 °C in an Eppendorf MasterCycler epGradientS (Eppendorf, Hamburg, Germany). A 3-μL aliquot of a 20 μM mix of primers IS7 and IS8 (Illumina) were added to the tubes after the elongation step and libraries were directly amplified by PCR. PCR was performed as follows: 95 °C for 2 min, followed by 9 amplification cycles (denaturation at 95 °C for 20 sec, primer annealing at 46 °C for 30 sec, and extension at 60 °C for 1.5 min). This amplification protocol was modified to reduce the amplification bias of GC and AT-rich DNA fragments ([46] and unpublished data). The purification of the libraries following nine cycles of amplification was carried out using 1.3× SPRI magnetic beads from the NucleoMag NGS clean-up and Size Select kit (Macherey Nagel, Düren, Germany). DNA was eluted with 50 μL of γ-irradiated H_2_O (irradiation with 2 kGy). This first eluate was purified a second time using the same protocol with SPRI magnetic beads and eluted in 30 μL of EB buffer. Amplified and purified libraries were visualized and quantified on a Bioanalyzer 2100 (Agilent, Santa Clara, CA) using a High Sensitivity DNA assay chip.

#### DNA sequence capture of mitochondrial genomes

For the production of complete mitogenome sequences, we developed a sequence capture approach using biotinylated RNA baits [47].

##### Capture: bait synthesis

The complete *Bos taurus* mitogenome was amplified using 11 overlapping PCR products (ca. 1.5 kb) containing the T3 RNA polymerase promoter sequence on the 5′ ends of either the forward or reverse strand. The 22 PCR reactions were performed in a final volume of 50 μL containing 12 ng of total genomic *B. taurus* DNA, 1 U of FastStart Taq, 1× FastStart buffer, 120 μM dATP, dCTP, dGTP and dUTP, 1 mg/mL BSA, and 0.4 μM of one of the 22 primer pairs (Additional file 1: Table S3). PCRs were performed on an Eppendorf MasterCycler epGradientS as follows: polymerase activation at 95 °C for 10 min, 45 amplification cycles (95 °C for 20 sec, 58 °C for 30 sec, 72 °C for 3 min), and a final extension step at 72 °C for 5 min. The amplification of each fragment was visualized by agarose gel electrophoresis in a 1 % agarose gel (100 volts for 30 min), and the products were purified using the QIAquick PCR purification kit. The purified mitochondrial fragments were then quantified on a Nanodrop ND-1000 spectrophotometer. The products were pooled in equimolar amounts in two different lots: one containing the fragments with the T3 RNA polymerase promotor sequence at the 5′ end of the forward strands, and the other with fragments containing the T3 RNA polymerase promotor sequence at the 5′ end of the reverse strands.

A total of 500 ng of each PCR product pool was used as a template in a 40 μL MEGAscript T3 transcription reaction (Ambion, Foster City, CA) containing 0.75 mM of each ATP, UTP and GTP, 0.375 mM of CTP, and 0.375 mM of Biotin-14-CTP (Invitrogen, Thermo Fisher Scientific). After an overnight incubation at 37 °C, the DNA template was digested with 2 U/40 μL reaction volume of TURBO DNase (Ambion) for 30 min at 37 °C. The ca. 1.5 kb transcripts were purified using an RNeasy MinElute Cleanup Kit (Qiagen) and eluted twice in 35 μL of nuclease-free water. The eluate was treated a second time with 5 U/40 μL reaction volume of Recombinant DNase1 (Roche) for 15 min at 35 °C in 1× Recombinant DNase1 buffer (Roche) in order to hydrolyze potentially remaining DNA template molecules. The DNase was removed through another purification of the transcripts using the RNeasy MinElute Cleanup Kit and the RNA was eluted as above. The purified ca. 1.5 kb transcripts were fragmented to generate RNA fragments ranging from 100 to 600 nucleotides with an average size of 300 nt using the NEBNext magnesium RNA Fragmentation Module kit (New England Biolabs) for 4 min at 94 °C. The fragmented transcripts were ethanol precipitated using three volumes of absolute ethanol and 0.3 M sodium acetate pH 5.5. The transcripts were washed with 500 μL of 70 % ethanol, dried and resuspended in 40 μL of nuclease-free water. The fragmented concentrated transcripts were visualized on a 2 % agarose gel, and the products were quantified using a Nanodrop ND-1000 spectrophotometer.

##### Capture: production of blocking oligos

PCR products corresponding to each N500 and N700 barcoded sequencing adaptor of the Nextera XT series (Illumina) containing the T7 RNA polymerase promotor sequence at the 5′ end of the forward strands were amplified in a 100 μL final volume reaction containing 100 pM of Nextera barcoded adaptor primer, 2 U FastStart Taq, 1× FastStart buffer, 120 μM of a mix of dNTPs, 1 mg/mL of BSA, and 0.5 μM of each N500 and N700 blocking oligo primers (N500 adaptor blocking oligos: T7BO + P5-F (5′ATGTAATACGACTCACTATAGGGAATGATACGGCGACCAC3′) and BO + P5-R (5′GGAAGAGCGTCGTGTAGG3′); N700 adaptor blocking oligos: T7BO + P7-F (5′ATGTAATACGACTCACTATAGGGAGATCGGAAGAGCACACG3′) and BO + P7-R (5′CAAGCAGAAGACGGCATAC3′). PCRs were performed on an Eppendorf MasterCycler epGradientS using a polymerase activation step at 95 °C for 10 min, followed by 25 amplification cycles (95 °C for 20 sec, 60 °C for 30 sec, 72 °C for 45 sec), and a final extension step at 72 °C for 2 min. PCR products were visualized on a 2 % agarose gel (100 volts for 1 hour), and then quantified using a Qubit 2.0 Fluorimeter. All PCR products were pooled in equimolar amounts and purified using the Qiagen Gel extraction protocol. DNA was eluted with 50 μL of nuclease-free water, and each purified pool was quantified using a Qubit 2.0 Fluorimeter; 50 ng of each PCR product pool were used as a template in a 20 μL MAXIscript T7 transcription (Ambion) containing 1 mM of each ATP, UTP, GTP, and CTP. After an overnight incubation at 37 °C, the DNA template was removed by a TURBO DNase treatment for 30 min at 37 °C. Unincorporated nucleotides, buffer components and DNase were removed by phenol-chloroform purification followed by alcohol precipitation. The blocking oligo transcript RNA was visualized on a 2 % agarose gel (100 volts for 1 hour), and quantified using a Nanodrop ND-1000 spectrophotometer.

##### Capture: hybrid selection

Each library was individually hybridized to the biotinylated probes in the presence of RNA blocking oligos complementary to the Illumina adapters for 48 hours at 62 °C. For each 38 μL final volume reaction, 15 μL of ancient DNA library (80–270 ng) was heated for 5 min at 95 °C, incubated for 5 min at 62 °C, and mixed with ca. 4 μg of each pool of blocking oligos (N500 or N700), prewarmed (62 °C) 2× hybridization buffer (10× SSPE, 10 mM EDTA and 0.2 % Tween-20), and ca. 500 ng prewarmed (2 min at 62 °C) mix of biotinylated RNA. After incubation, the hybridization mix was added to 500 ng (50 μL) M-280 streptavidin Dynabeads (Invitrogen), that had been washed three times and resuspended in 162 μL 1 M NaCl, 10 mM Tris-HCl, pH 7.5, 1 mM EDTA, and 1× Denhardt’s solution. After 40 min at room temperature, the beads were pulled down and washed once at room temperature for 15 min with 0.2 mL 1× SSC/0.1 % Tween-20, followed by three 10 min washes at 62 °C with 0.2 mL 0.1× SSC/0.1 % Tween-20, resuspending the beads once at each washing step. Hybrid-selected DNA was eluted twice with 50 μL 0.1 N NaOH. After 10 min at 20 °C, the beads were pulled down and the supernatant transferred to a tube containing 75 μL 1 M Tris-HCl, pH 7.5. The captured single stranded DNA fragments were then purified using the Qiagen Gel extraction protocol. DNA was eluted with 30 μL of EB buffer.

##### Capture: PCR amplification of enriched library

Amplification of the enriched library was performed using 25 μL of each library and FastStart Taq with 1× FastStart buffer, 100 μM each of dNTPs, and 0.4 μM of each Illumina primer IS7 and IS8 in a 50 μL final volume on an Eppendorf MasterCycler epGradient S. After an 8 min activation of the FastStart Taq at 95 °C, PCR was carried out for the appropriate number of cycles (95 °C for 20 sec, 46 °C for 30 sec, and 60 °C for 2 min, followed by a 2 min extension) to avoid plateau, as determined by a previous qPCR under similar conditions using 1 μL of each captured library in 10 μL total volume for 30 cycles. Amplified enriched libraries were then purified using the QIAquick PCR purification kit and quantified on a Nanodrop spectrophotometer.

A second round of capture was then performed as above, on all amplified and purified enriched libraries. After purification and amplification, the double-enriched libraries were then purified using 1.3× SPRI magnetic beads from the NucleoMag NGS clean-up and Size Select kit. DNA was eluted with 50 μL of EB buffer, and the eluates were purified a second time using the SPRI magnetic beads protocol with a final elution in 30 μL of EB buffer. The double-enriched amplified and purified libraries were visualized and quantified on a Bioanalyzer 2100 using a High Sensitivity DNA assay chip.

#### Sequencing

Enriched purified libraries were pooled in equimolar amounts, and paired-end sequenced using 2 × 75 cycles on a V3 flowcell for MiSeq Illumina platform at the Jacques Monod Institute (Paris, France) according to the manufacturer’s protocol.

### Bioinformatic processing and mapping of the reads

Paired-end reads were merged using leeHom using the --ancientdna parameter [48]. Merged reads were then mapped to modern *Bison bison* and *Bison bonasus* mitogenomes using bwa as described previously [37]. The first 100 nt of each linearized reference mitogenome used for mapping was duplicated at the 3′ end to allow mapping of fragments overlapping the junction. Mapped read duplicates were then removed using samtools rmdup as previously described [37]. The resulting bam files were then imported into Geneious 6.1.8 [43] and remapped onto the appropriate mitogenome sequence without the 100 nt duplication (*B. bison* for *B. priscus* sequences, *B. bonasus* for the Bb1 and Bb2 sequences). Consensus sequences were generated in Geneious and verified by visual inspection of the aligned reads. Geneious was used to measure coverage depth and the number of covered bases displayed in Additional file 1: Table S4.

### Phylogenetic analyses

Sequence alignments were performed using the Muscle algorithm and were visually inspected and adjusted using Geneious 6.1.8 [43]. The maximum likelihood analyses presented in Fig. 1 were computed using PHYML 3.0, using an HKY substitution model with a gamma-distributed rate of variation among sites (+G) and invariant sites (+I) [49]. Robustness of the nodes was estimated using 500 bootstraps. RaXML 8.2.3 was used to generate the maximum likelihood bootstrap support values for the complete mitogenome alignment shown in Fig. 3 [50].

Phylogenetic analyses conducted under the Bayesian framework were performed using the program BEAST v. 1.8.2, which allows estimation of mutation and population history parameters simultaneously from temporally spaced sequence data [51]. Nucleotide substitution models were chosen following comparisons performed with jModelTest 2.1.7 using the Bayesian Information Criteria [52]. The HVR analysis presented in Fig. 2 was performed considering a TN93 model for the nucleotide substitution model, a gamma-distributed rate of variation among sites (+G) with four rate categories and invariant sites (i.e., TN93 + I + G model). For the complete mitogenome analysis presented in Fig. 3, we used four partitions, the HVR, the first and second positions of the codons within the coding region, the third position, and the RNA genes. We considered the HKY + I + G model for the first two partitions, and the TN93 + G for the last two. Default priors were used for all parameters of the nucleotide substitution model. For the analysis of Fig. 2, we used a strict molecular clock with a lognormal prior for the substitution rate (mean = –15.0, stdev = 1.4) corresponding to a median of 2 × 10^–7^ substitutions per site and per year (95 % HPD 1.3 × 10^–8^ to 3.2 × 10^–6^) based on the estimation for Bison HVR substitution rate [7]. For the various partitions of the mitogenome of Fig. 3, we used estimates for the human mitogenome substitution rate to set the priors [53]: lognormal priors: HVR, mean = –16.1 stdev = 2.0, corresponding to a median of 1.0 × 10^–7^ (2 × 10^–9^ to 5 × 10^–6^); RNA, mean = –18.65 stdev = 2.0, corresponding to a median of 8.0 × 10^–9^ (1.6 × 10^–10^ to 4 × 10^–7^); first and second positions, mean = –18.5 stdev = 2.0, corresponding to a median of 9.0 × 10^–9^ (1.8 × 10^–10^ to 4.7 × 10^–7^); third position, mean = –17.7 stdev = 2.0 corresponding to a median of 2.0 × 10^–8^ (4× 10^–10^ to 1 × 10^–6^). Finally, a standard coalescent model was considered for the tree prior with a Bayesian skyline plot to model populations (5 and 10 populations with default parameters for the HVR and the mitogenomes respectively). The prior for the tree height followed a log-normal distribution, mean = 14.6 stdev = 0.6, truncate to 8.0 × 10^6^ and 1.0 × 10^5^, corresponding to a median of 2.2 × 10^6^ and a 95 % HPD of (6.3 × 10^6^ to 6.7 × 10^5^), which integrates the various fossil finds assumed to correspond to ancestors of cattle and bison [54, 55].

To estimate the posterior distribution of each parameter of interest, we used the Markov Chain Monte Carlo algorithm implemented in the BEAST software. We ran five independent chains with initial values sampled as described above and an input UPGMA tree constructed using a Juke-Cantor distance matrix. Each of these chains was run for 50,000,000 iterations and for each parameter of interest, 18,000 samples (one every 2500 generated ones) were drawn after discarding a 10 % burn-in period. The BEAST output was analyzed with the software Tracer v. 1.6 (http://tree.bio.ed.ac.uk/software/tracer/). Visual inspection of the traces and the estimated posterior distributions suggested that each MCMC had converged on its stationary distribution. Using Logcombiner v. 1.8.2, we further combined all the results from the five independent chains. The maximum clade credibility tree with the median height of the nodes was finally calculated using TreeAnnotator v. 1.8.2 and visualized using FigTree v. 1.4.2 (http://tree.bio.ed.ac.uk/software/figtree/).

## Additional files

References cited in the Additional files 1, 2, 3, 4, 5, and 6: [56–77] Additional file 1: Table S1. Description of all samples analyzed. **Table S2.** Population diversity parameters based on the HVR sequences. **Table S3.** GenBank accession numbers of the sequences used in the Bayesian analysis of the HVR phylogeny of Fig. 2. **Table S4.** Depth of coverage and number of covered positions of the captured mitogenomes. **Table S5.** GenBank accession numbers of the sequences used in the Bayesian analysis of the mitogenome phylogeny of Fig. 3. **Table S6.** PCR primers used for amplification of the mitochondrial HVR DNA of *Bison sp*. **Table S7.** Primer sequences for amplification of the mitochondrial genome of *Bos taurus* to synthesize capture baits. (XLSX 177 kb)
Additional file 2: Figure S1. Alignment of the HVR sequences obtained in the present study. Alignment of the sequences originating from the samples listed in Table S1 using the primers listed in Additional file 1: Table S6. (PPTX 131 kb)
Additional file 3: Figure S2. Schematic representation of the two hypotheses describing the mtDNA segregation pattern between *B. p taurus*, *B. bonasus*, and *B. priscus/bison*. Species tree and embedded mtDNA tree are shown with the age estimate of the two nodes and the 95 % HPD bar are represented to scale using the values estimated with the Bayesian analyses of the complete mitogenomes presented in Fig. 3. Hypothesis 2 of the post-speciation gene flow requires a rapid speciation event taking place just during the short time-interval between the two nodes. This hypothesis appears less parsimonious because speciation is clearly gradual since it has not yet completely prevented interfertility between *Bos* and *Bison* even 800 kyr after the hypothesized rapid speciation event. (PPTX 45 kb)
Additional file 4: Figure S3. Distribution of the SNP density along the mitogenome for sequences analyzed in this study. Each bison mitogenome was aligned pairwise to the same outgroup, the bovine mitogenome reference sequence. The quantity of SNPs in 50 bp-long sliding windows (step of 5 bp) is plotted in ordinate alongside the length of the mitogenome in abscissa. Protein and RNA coding genes, and the HVR are schematized below in green, purple and pink, respectively. (PPTX 271 kb)
Additional file 5: Document S1. Description of the samples that yielded genetic results and of their sites of origin. (DOCX 761 kb)
Additional file 6: Figure S4. Map showing the location of the various sites in Western Europe that yielded the samples described in this study. (PPTX 1296 kb)
