# Supplementary material for: Past climate changes, population dynamics and the origin of Bison in Europe
Source: BMC Biol. 2016 Oct 21;14:93. doi: 10.1186/s12915-016-0317-7 (PMC5075162; doi:10.1186/s12915-016-0317-7)
Supplement: Additional file 2: Figure S1. — Alignment of the HVR sequences obtained in the present study. Alignment of the sequences originating from the samples listed in Table S1 using the primers listed in Additional file 1: Table S6. (PPTX 131 kb) [file 12915_2016_317_MOESM2_ESM.pptx]

## Slide 1
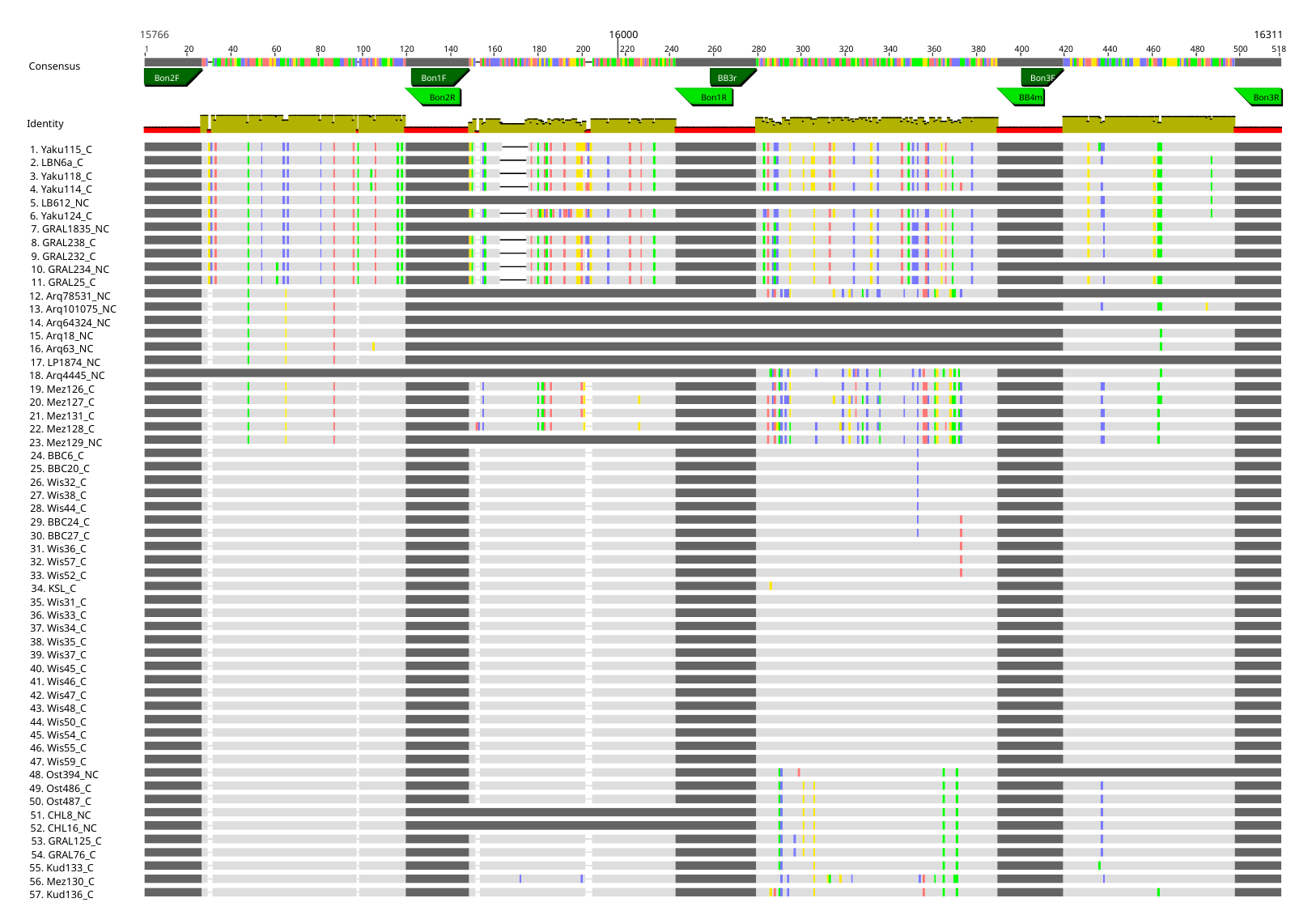

15766
16000
16311
1
20
40
60
Bon2F
Bon1F
BB3r
Bon3F
Bon2R
Bon1R
BB4m
Bon3R
80
100
120
140
160
180
200
220
240
260
280
300
320
340
360
380
400
420
440
460
480
500
518
Consensus
Identity
1. Yaku115_C
2. LBN6a_C
3. Yaku118_C
4. Yaku114_C
5. LB612_NC
6. Yaku124_C
7. GRAL1835_NC
8. GRAL238_C
9. GRAL232_C
10. GRAL234_NC
11. GRAL25_C
12. Arq78531_NC
13. Arq101075_NC
14. Arq64324_NC
15. Arq18_NC
16. Arq63_NC
17. LP1874_NC
18. Arq4445_NC
19. Mez126_C
20. Mez127_C
21. Mez131_C
22. Mez128_C
23. Mez129_NC
24. BBC6_C
25. BBC20_C
26. Wis32_C
27. Wis38_C
28. Wis44_C
29. BBC24_C
30. BBC27_C
31. Wis36_C
32. Wis57_C
33. Wis52_C
34. KSL_C
35. Wis31_C
36. Wis33_C
37. Wis34_C
38. Wis35_C
39. Wis37_C
40. Wis45_C
41. Wis46_C
42. Wis47_C
43. Wis48_C
44. Wis50_C
45. Wis54_C
46. Wis55_C
47. Wis59_C
48. Ost394_NC
49. Ost486_C
50. Ost487_C
51. CHL8_NC
52. CHL16_NC
53. GRAL125_C
54. GRAL76_C
55. Kud133_C
56. Mez130_C
57. Kud136_C
