# Supplementary material for: Past climate changes, population dynamics and the origin of Bison in Europe
Source: BMC Biol. 2016 Oct 21;14:93. doi: 10.1186/s12915-016-0317-7 (PMC5075162; doi:10.1186/s12915-016-0317-7)
Supplement: Additional file 3: Figure S2. — Schematic representation of the two hypotheses describing the mtDNA segregation pattern between B. p taurus, B. bonasus, and B. priscus/bison. Species tree and embedded mtDNA tree are shown with the age estimate of the two nodes and the 95 % HPD bar are represented to scale using the values estimated with the Bayesian analyses of the complete mitogenomes presented in Fig. 3. Hypothesis 2 of the post-speciation gene flow requires a rapid speciation event taking place just during the short time-interval between the two nodes. This hypothesis appears less parsimonious because speciation is clearly gradual since it has not yet completely prevented interfertility between Bos and Bison even 800 kyr after the hypothesized rapid speciation event. (PPTX 45 kb) [file 12915_2016_317_MOESM3_ESM.pptx]

## Slide 1
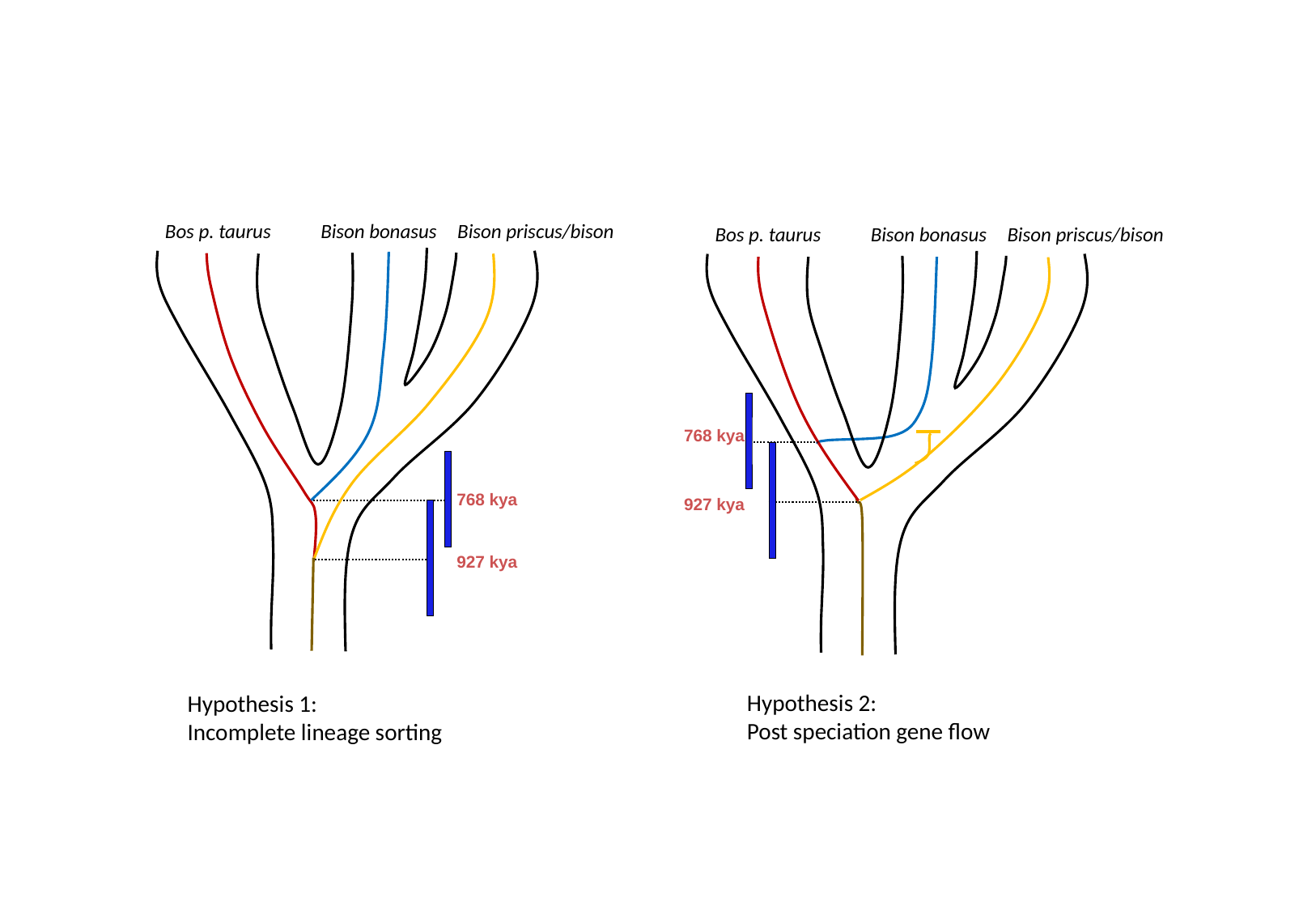

Bos p. taurus
Bison bonasus
Bison priscus/bison
Bos p. taurus
Bison bonasus
Bison priscus/bison
768 kya
768 kya
927 kya
927 kya
Hypothesis 2:
Post speciation gene flow
Hypothesis 1:
Incomplete lineage sorting
