# Supplementary material for: Past climate changes, population dynamics and the origin of Bison in Europe
Source: BMC Biol. 2016 Oct 21;14:93. doi: 10.1186/s12915-016-0317-7 (PMC5075162; doi:10.1186/s12915-016-0317-7)
Supplement: Additional file 4: Figure S3. — Distribution of the SNP density along the mitogenome for sequences analyzed in this study. Each bison mitogenome was aligned pairwise to the same outgroup, the bovine mitogenome reference sequence. The quantity of SNPs in 50 bp-long sliding windows (step of 5 bp) is plotted in ordinate alongside the length of the mitogenome in abscissa. Protein and RNA coding genes, and the HVR are schematized below in green, purple and pink, respectively. (PPTX 271 kb) [file 12915_2016_317_MOESM4_ESM.pptx]

## Slide 1
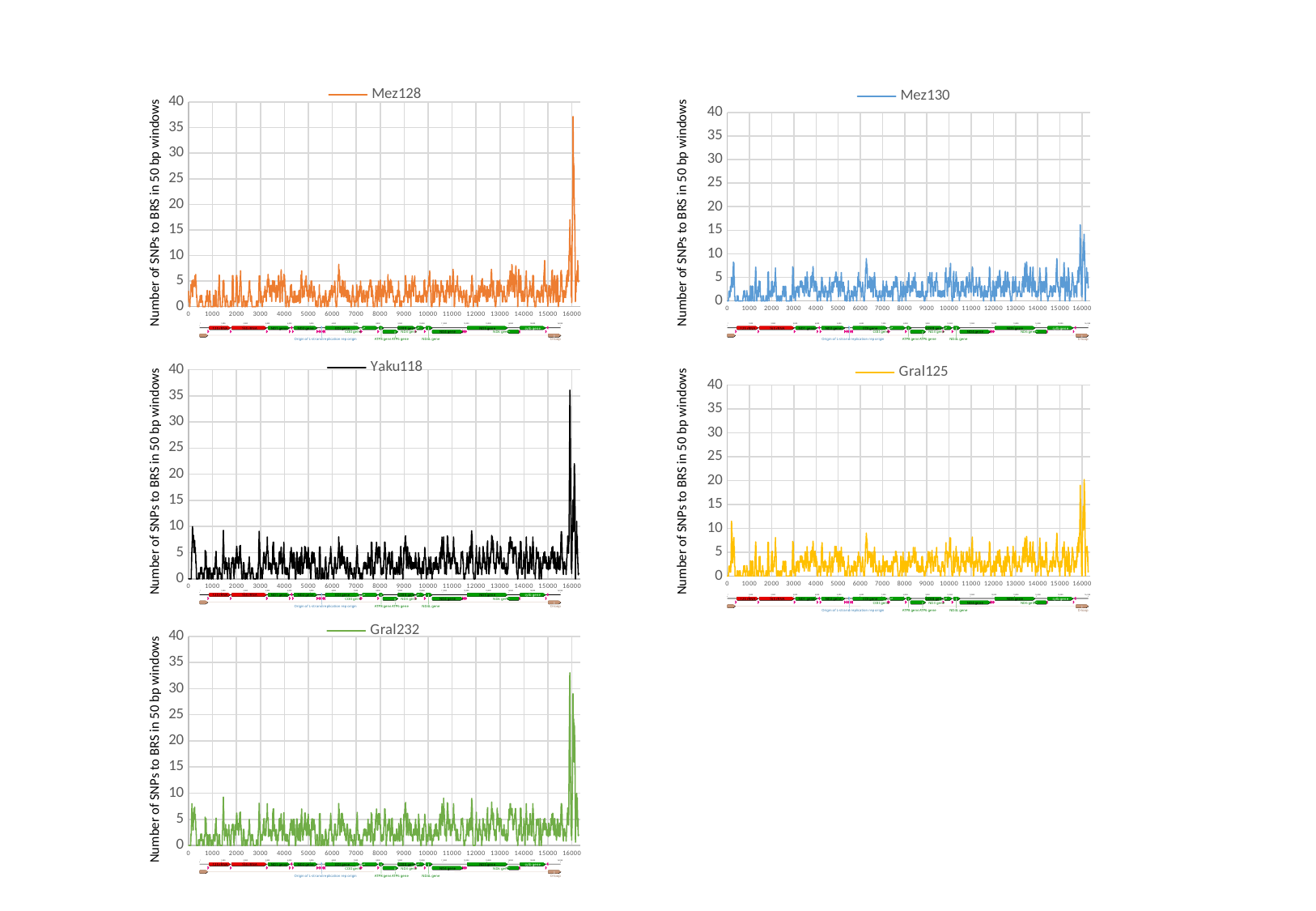

### Chart
| Category | |
|---|---|
### Chart
| Category | |
|---|---|Number of SNPs to BRS in 50 bp windows
Number of SNPs to BRS in 50 bp windows
1
1,000
2,000
3,000
4,000
5,000
6,000
7,000
8,000
9,000
10,000
11,000
12S rRNA
16S rRNA
ND1 gene
ND2 gene
COI gene
COIII gene
ND5 gene
cytb gene
COII gene
ND3 gene
ND4 gene
ND6 gene
Origin of L-strand replication rep origin
ATP8 gene
ATP6 gene
ND4L gene
D-loop
12,000
13,000
14,000
15,000
16,338
1
1,000
2,000
3,000
4,000
5,000
6,000
7,000
8,000
9,000
10,000
11,000
12S rRNA
16S rRNA
ND1 gene
ND2 gene
COI gene
COIII gene
ND5 gene
cytb gene
COII gene
ND3 gene
ND4 gene
ND6 gene
Origin of L-strand replication rep origin
ATP8 gene
ATP6 gene
ND4L gene
D-loop
12,000
13,000
14,000
15,000
16,338
### Chart
| Category | |
|---|---|
### Chart
| Category | |
|---|---|Number of SNPs to BRS in 50 bp windows
Number of SNPs to BRS in 50 bp windows
1
1,000
2,000
3,000
4,000
5,000
6,000
7,000
8,000
9,000
10,000
11,000
12S rRNA
16S rRNA
ND1 gene
ND2 gene
COI gene
COIII gene
ND5 gene
cytb gene
COII gene
ND3 gene
ND4 gene
ND6 gene
Origin of L-strand replication rep origin
ATP8 gene
ATP6 gene
ND4L gene
D-loop
12,000
13,000
14,000
15,000
16,338
### Chart
| Category | |
|---|---|
1
1,000
2,000
3,000
4,000
5,000
6,000
7,000
8,000
9,000
10,000
11,000
12S rRNA
16S rRNA
ND1 gene
ND2 gene
COI gene
COIII gene
ND5 gene
cytb gene
COII gene
ND3 gene
ND4 gene
ND6 gene
Origin of L-strand replication rep origin
ATP8 gene
ATP6 gene
ND4L gene
D-loop
12,000
13,000
14,000
15,000
16,338
Number of SNPs to BRS in 50 bp windows
1
1,000
2,000
3,000
4,000
5,000
6,000
7,000
8,000
9,000
10,000
11,000
12S rRNA
16S rRNA
ND1 gene
ND2 gene
COI gene
COIII gene
ND5 gene
cytb gene
COII gene
ND3 gene
ND4 gene
ND6 gene
Origin of L-strand replication rep origin
ATP8 gene
ATP6 gene
ND4L gene
D-loop
12,000
13,000
14,000
15,000
16,338
