# Supplementary material for: Past climate changes, population dynamics and the origin of Bison in Europe
Source: BMC Biol. 2016 Oct 21;14:93. doi: 10.1186/s12915-016-0317-7 (PMC5075162; doi:10.1186/s12915-016-0317-7)
Supplement: Additional file 5: Document S1. — Description of the samples that yielded genetic results and of their sites of origin. (DOCX 761 kb) [file 12915_2016_317_MOESM5_ESM.docx]

**Supporting Information. Massilani et al. European bison history since the late Pleistocene.**

**Description of the samples that yielded genetic results and of their sites of origin.**

The 85 samples that have been analyzed genetically are listed in **Table S1** in a separate Excel file. Those that yielded genetic results are described herein together with the archeological, paleontological or collection site they originate from.

- 1. **Mezmaiskaya cave – Northern Caucasus**

*Gennady Baryshnikov*

Mezmaiskaya Cave lies 1310 m a.s.l., in the Northern Caucasus, Russia. It is 35m deep, and up to 25m wide. The site was discovered and first excavated by L. V. Golovanova between 1987 and 2003 ^1^. In addition to two Neanderthal infants, the excavators found many remains of medium and large mammals, providing considerable material for ESR dating. Faunal material is remarkably unweathered, and, along with extensive rodent and pollen remains, allows estimation of the climate throughout the stratigraphy. In additon to bovids, the cave contains *Ursus spelaeus*, some bird, rodent, and carnivore remains. The majority of the bovids, cervids, caprids, and other ungulates represent human kills of prime adults ^2^.

The 6 bones from Mezmaiskaya cave analyzed in the present study were collected from level 2b between 1988 and 1989 and then conserved at the Zoological Institute, Russian Academy of Sciences in St. Petersburg. Details are presented in Table S8. The dating we performed confirms the accuracy of the age of layer 2b described previously ^3^.

**Table S8: Samples of the Mezmaiskaya cave.**

| Sample ID | Location | Bone type | Year of acquisition | Layer | Age (cal kyr BP) |
| --- | --- | --- | --- | --- | --- |
| 126 | Mezmaiskaya cave | humerus | 1988 | 2b | ND |
| 127 | Mezmaiskaya cave | scapula | 1988 | 2b | 46.5-44.6 |
| 128 | Mezmaiskaya cave | skull | 1988 | 2b | ND |
| 129 | Mezmaiskaya cave | long bone | 1989 | 2b | 47.9-45.5 |
| 130 | Mezmaiskaya cave | scapula | 1989 | 2b | 51.0-47.7 |
| 131 | Mezmaiskaya cave | rib | 1988 | 2b | ND |

The obtained radiocarbon dates are compared in supplementary table 3 with those previously published ^3^. ND=not determined

**Table S9: ^14^C Radiocarbon dating of Mezmaiskaya cave layers. From ^3^**

| **Layer** | **Laboratory number** | **Material** | **Age (kyr BP)** |
| --- | --- | --- | --- |
| 1C | Beta-113536 | Wood charcoal | 32.0±0.3 |
| 2 | LE-4735 | Bone | 32.2±0.7 |
| 2A | Beta-53896/CAMS-2999 | Burnt bone | 35.8±0.4 |
| 2A | Beta-53897/ETH-9817 | Burnt bone | 36.3±0.5 |
| 2B | LE-3599 | Bone | 40.7±1.6 |
| 3 | UA-14512 | Neanderthal rib | 29. ±1.0 |
| 3 | LE-3841 | Bone | >45.0 |

- 1. **Kudaro 1 and 3 caves - Southern Caucasus**

*Gennady Baryshnikov*

Kudaro 1 and Kudaro 3 caves are situated in the central part of the southern slope of the Greater Caucasus. They are placed one above another on the left bank of the Djedjori River (Rioni River basin) near Kvaisa City in Southern Ossetia ^4^. They represent gallery-type caves and are situated at a elevation of 1,600 m above sea level. The site was discovered and has been excavated regularly since 1956 ^4^.

Loamy clay deposits of the caves contain archaeological artifacts of Acheulean, Mousterian, and more recent periods. The Mousterian layer 3a has been radiocarbon dated at 44.150±2.400/1.850 (Gr-6079) ^5^, as well as cave bear remains from layer 3 have three AMS dates: >41,600 (OxA-19611), 47,900±2,500 (Ox-19612) and 47,700±1,800 (OxA-19613)^,6^. The analyzed samples that had been conserved at the Zoological Institute, Russian Academy of Sciences in St. Petersburg are shown in table S10.

**Table S10: Samples of the Kudaro cave.**

| Sample ID | Location | Bone type | Year of acquisition | Layer | Age (cal kyr BP) |
| --- | --- | --- | --- | --- | --- |
| 132 | Kudaro 3 | mandible | 1977 | 2 | ND |
| 133 | Kudaro 3 | mandible | 1977 | 2 | ND |
| 134 | Kudaro 3 | mandible | 1977 | 2 | 0.1 (1909) |
| 135 | Kudaro 3 | mandible | 1977 | 2 | 0.1 (1911) |
| 136 | Kudaro 1 | metacarpal | 1984 | 3ab-2 | ND |
| 137 | Kudaro 1 | metacarpal | 1984 | 3ab-2 | 0.7 (1949) |

- 1. **Yakutia** (Northeast of Siberia, Russia)

*Gennady Boeskorov*

Samples from Yakutia (Northeast of Siberia) from the lower stream of the Kolyma River belong to Pleistocene bison (*Bison priscus*) and were curated in the Geological Museum of Institute of Diamond and Precious Metals Geology, Russian Academy of Sciences, Yakutsk, Russia.

Yaku114 is a bison mandible fragment from the Lower Kolyma region, vicinity of Chersky village, Malaya Filippova river (about 7 km N-E from Chersky). Yaku118 is a bison skull fragment from the Lower Kolyma region in the vicinity of Chersky village, Malaya Filippova river (about 7 km N-E from Chersky). Both specimens were found by Gennady Boeskorov and Sergey Davidov in September 2009.

These bones probably belong to the Sartanian glacial or Karginian interstadial (period of warming) of the Late Pleistocene, because some bones from Malaya Filippova locality belong to that annual interval: one *Bison priscus* bone from that locality was dated to 22,280±310 yBP (Institute of Geology and Minerology, Novosibirsk SOAN-7585); a molar of the woolly mammoth *Mammuthus primigenius* was dated to 24,820±310 kya (Institute of Geology and Minerology, Novosibirsk SOAN-7586); a rib fragment of a mummified cadaver of the woolly rhinoceros (Coelodonta antiquitatis Blum., 1799) from that locality was dated by AMS-radiocarbon method to 39,140±390 years ago (OxA-18755) ^7^.

Yaku115 is a bison skull fragment from the Lower Kolyma region, vicinity of Chersky village, Pervyi ruchey (1^st^ stream), and was found in the 2000s. The skull belongs to an adult bull of a large size (condilobasal length of skull is 620 mm, length of the horn sheath on outer curvature is 910 mm). The skull is kept in the collections of Museum of Nature in Chersky village (no collection number); no radiocarbon dating, likely belonging to the Late Pleistocene.

Yaku124 is a bison horn sheath from the Lower Kolyma region, Drevniy stream (about 25 km East from Chersky village. It was found by Gennady Boeskorov and Sergey Davidov in September 2010. It probably belongs to the Karginian interstadial of Late Pleistocene, because two *Bison priscus* bones from that locality were dated to 26,200±370 yBP (Institute of Geology and Minerology, Novosibirsk SOAN-7583) and 39,450±460 yBP (Institute of Geology and Minerology, Novosibirsk SOAN-7836) and belong to that interstadial.

- 1. **La Berbie (Dordogne, France)**

*Stéphane Madelaine*

La Berbie (Castels, Dordogne) is a cave/sink-hole filled with fossils that yielded around 5,000 bones distributed over a stratigraphy of 10 meters ^8,9^. The steppe bison *Bison priscus* constitutes the majority of the remains, with 60 % of the remains and a minimum of 30 individuals. The other species present are diverse and in decreasing order reindeer, horse, chamois, megaceros, deer, mammoth, woolly rhinoceros, cave hyena, fox, wolf, polecat, hare, jackdaw, eagle. Three conventional radiocarbon dates yielded 35,830 +/- 500 yrs BP (Gif A98104) at the level of the underlying cave at 15 m depth, 33,490 +/ 250 yrs BP (Gif 10545) and 33,220 +/- 270 yrs BP (Gif 9994) for the fill-in of the sink-hole at 4 meters difference suggesting that the deposition was rapid during a cold period of the isotopic stage 3. These three dates were obtained directly on bison bones.

Despite a few lithic artefacts and the presence of cave hyenas, the taphonomic study suggests accidental fall as main or only cause of this skeletal accumulation ^10^.

- 1. **Igue du Gral**

*Jean-Christophe Castel*

The paleontological site of the Igue du Gral (Sauliac-sur-Célé, Lot**)** is a sink-hole on the Jurassic plateau of the Quercy at 300 m .a.s.l., 170 m above the Lot and the Célé valley ^11-13^. The filling has been excavated over 2 m depth yielding several horizontal levels with rich and well preserved fauna. Radiocarbon dates were obtained from around 50 skeletal remains. The dates are between 10,440 yrs BP and 31990+/-240 years BP for the excavated sequence, covering the end of the MIS3 and the MIS2 ^14^. More than 26,000 macro- and mesofaunal remains have been determined and the principal mammal species are (in decreasing abundance): reindeer, Leporidae, horse, bison, wolf, deer, fox, mustelids, bear, ibex, chamois, saiga antelope, mammoth, rhinoceros, wolverine. Thus, the sequence is characterized by a cold paleoenvironment with a steppic faunal association, except for the upper sequence where deer and bison indicate a milder climate.


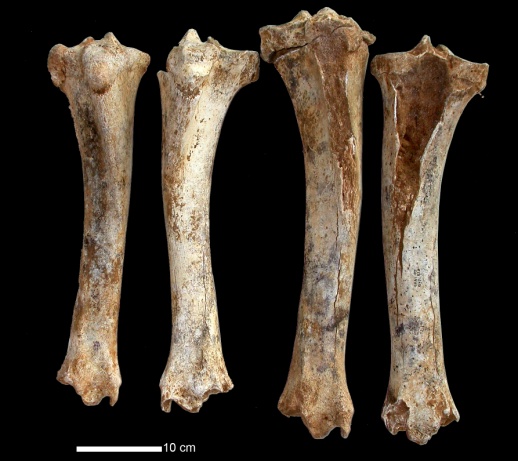
Six of the analyzed bison bones have been dated to 29,160 +/-180, 28,530 +/- 460, 26,700 +/- 190, 19,290 +/- 150, 10,260 ± 50 and 10,180 +/-40 years BP, but only the 19,290, 10,260 and 10,180 year-old specimens yielded genetic results. Moreover, the analysis of the DNA from four tibias from level 3 was successful, this level being dated on other bone remains to 14,355+/-995 years BP.


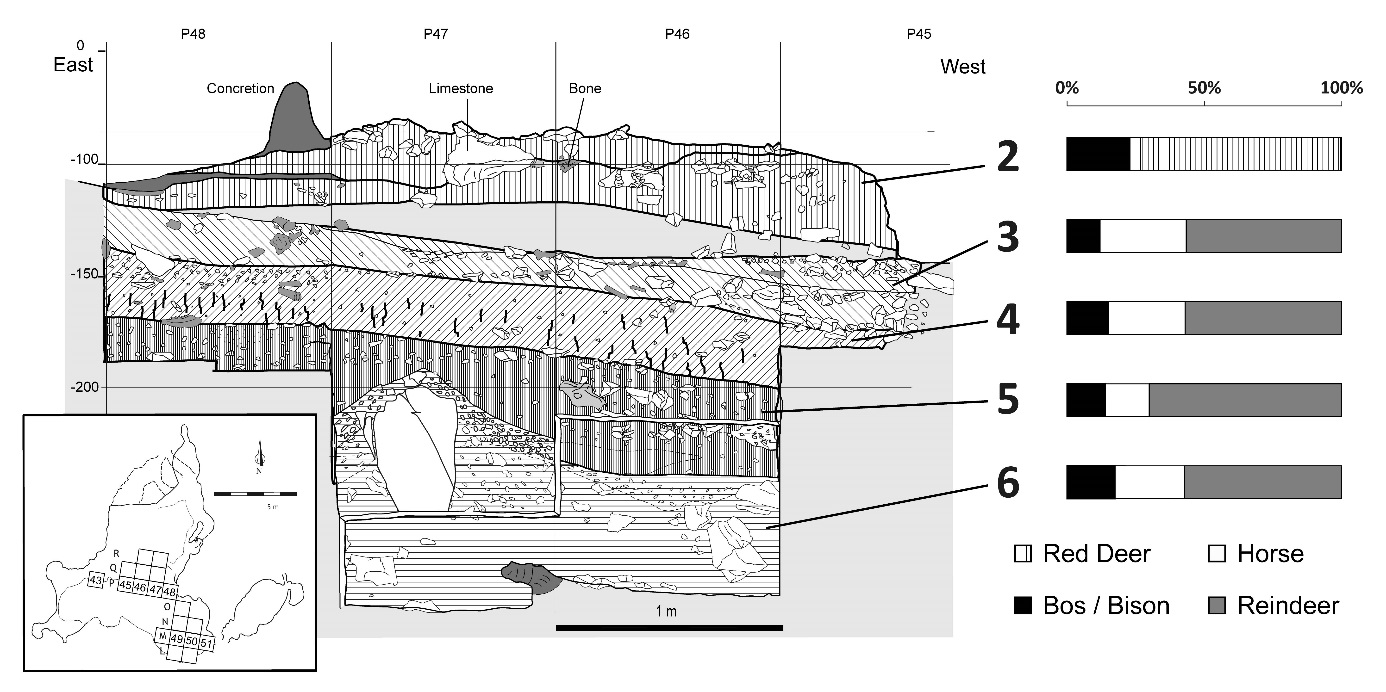


**Figure S5: Stratigraphy of the cave Igue du Gral** ^14^**.**

- 1. **L’Aven de l’Arquet (Ardèche, France)**

*Jean-Philip Brugal*

Arquet is located in south-east France (Ardéche) on the right bank on the Rhone Valley, on a limestone plateau at 340 m a.s.l. ^15^. It is a 27m deep karstic sink-hole which was a trap pit during the Late Pleistocene. More than 100,000 fossil bones and teeth are distributed between -10 m and -22 m and were radiocarbon-dated between ca. 42-30 kya (second part of MIS 3) covering several varying short climatic phases. This deposit has been excavated by spelunkers and most of the material has an imprecise stratigraphic position. The large mammal association is very rich (MNI of 537) and diversified, with overall 11 carnivore species (mainly wolf and red fox) and 10 herbivore species (in decreasing abundance: bison, reindeer, red deer and horse), as well as many micro-vertebrates. Few human remains (teeth) and lithic artifacts have been found, representing Middle Paleolithic and Neanderthals as well as Late Paleolithic and modern humans. The bison, attributed to the steppe bison *Bison priscus*, represent 127 individuals of both sexes with 36% of one to two year-old juvenile individuals. This bison bone assemblage represents probably one of the biggest fossil bison series in Western Europe. All skeletal parts are present and most of the bones are complete and relatively well preserved. Skulls are fragmented yielding horn-core fragments, more or less complete tooth-rows, isolated teeth and hard parts, such as petrosal bones. Eleven of these petrosal bones were analyzed here paleogenetically.

- 1. **Les Plumettes (Lussac les Châteaux, Vienne, France)**

*Stéphane Madelaine, Jean-Philippe Brugal,*

This small karstic cave-site (40 m²) yielded some lithic Mousterian industry of the Post-Quina or final Mousterian type associated with a rich and diversified fauna ^16^. The faunal association is characteristic of open and more forested areas and is composed of herbivores (mainly horse and bison, but also hydruntine, woolly rhino, mammoth, red deer, reindeer, megaceros, boar, etc.) and carnivores (cave hyena dominant, lion, cave bear, wolf, fox, etc.) ^17^. The site was attributed to the ‘Wurmian interstadial’ ^18^, first part of MIS3, and interpreted as a hyena den site occupation with limited human activities. Two ^14^C dates on giant deer bones yielded 40,600+/-1,200 (OxA-23085, level II) and 47,700+/-3,300 (OxA-23086, level IV) years BP ^19^.

- 1. **Kesslerloch**

*Hans-Peter Uerpmann*

Kesslerloch Cave was occupied in the Oldest Dryas, from approximately 15,500 to 12,300 years calBC. A dog maxilla has been AMS-dated in the Leibnitz-laboratory in Kiel (KIA-33350) to 12,225±45 years BP, which calibrates to 12,360±210 years calBP (CalPal). Of the 16 dates that span the main occupation phase of Kesslerloch, the dog maxilla delivered the youngest date. It falls just within the last few centuries before the onset of the late glacial interstadial complex (Bølling/Allerød). The occupation seems to terminate with the environmental changes at the beginning of this rewarming. The analyzed bone was radiocarbon dated to 12,180 ± 70 years BP.

- 1. **Chalain (Franche-Compté, France)**

*Rose-Marie Arbogast*

The lakes of Chalain and Clairvaux are located in the Combe d’Ain, an enclosed alluvial valley, at an altitude of 500 m a.s.l., within the plateau of the French Jura in the Franche-Comté. The lacustrine Neolithic sites at the lake of Chalain have chronological archaeological sequences dating from 3,850 to 850 BCE ^20^. The lake is located at 500 m a.s.l. constituting the upper limit of the extension of cereal agriculture and presents therefore an example of a particular adaptation of Neolithic civilisations to a rough climate. The archaeological villages of Chalain are not spread around the periphery of the lake but rather located at the West of the lake where they are separated from the banks of the lake by a swampy belt ^20,21^. The bones are exceptionally well preserved, presumably because the archaeological objects and bones were quickly covered by a layer of lacustrine chalk and preserved under anoxic conditions, either covered by water or below the ground water. Two skeletal remains, CHL8 and CHL16, have been included in the present study.

- 1. **Ostheim Birgelsgaerten (France)**

*Olivier Putelat*

The late medieval site of Ostheim « Birgelsgaerten » (7^th^ -8^th^ c. CE) yielded more than 3,000 animal remains. Their study ^22^, still underway, shows a large quantity of game in the excavated cabin 3219 and in the ditch 3325, which are located outside of the enclosure that limits this rural establishment, in contrast to the rarity of game remains in the rest of the site. Apart from the domestic faunal spectrum that is typical for Merovingian Alsace, a high ratio of wild faunal remains were found (10,5% of 808 determined remains and 14% of the weight of the remains). The small wild fauna is typical for the humid environment that characterizes « Birgelsgaerten » (otter, wild duck, fishes). Moreover, four different types of deer, wild boar and, exceptionaly, aurochs bones (a calcanéus and probably also a coxal), European bison (a coxal, a roximal phalangx) and elk (various skeletal parts). Three samples have been included in the present study that had been assigned to either *B. primigenius* (Ost 394) or *B. bonasus* (Ost 487) or either of the two species (Ost 486).

| Specimen | code | Assignment | Genetic result | photo |
| --- | --- | --- | --- | --- |
| 5006-OFA-3325-394 | Ost394 (B) | aurochs | bison | 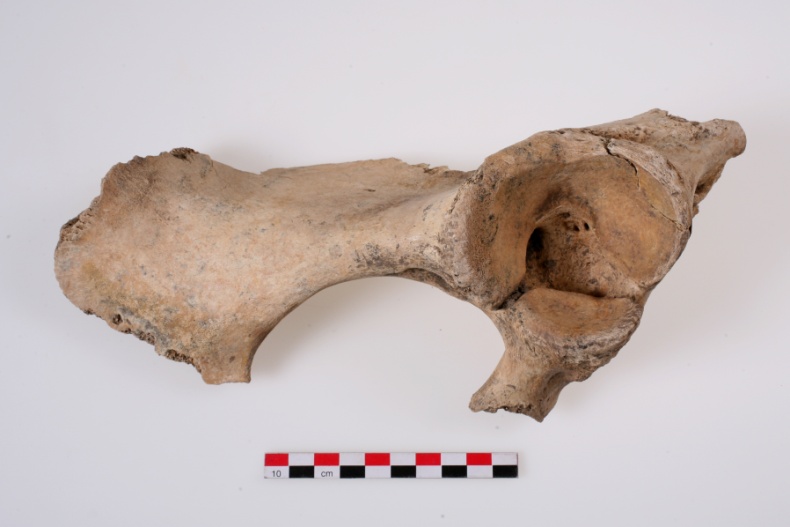 |
| 5006-OFA-3325.1 US2-0487/4857-OFA-3325-2 | Ost487 (D) | bison | bison | 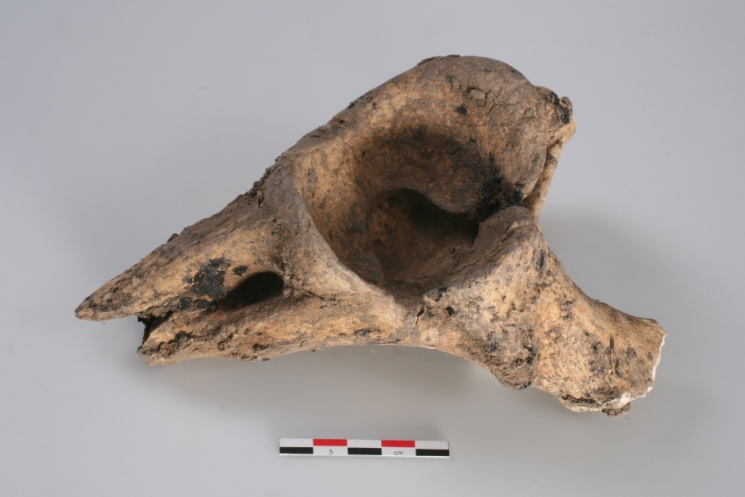 |
| 5006-OFA-3325.1-US2-0486 | Ost486(C ) | aurochs/bison | bison | 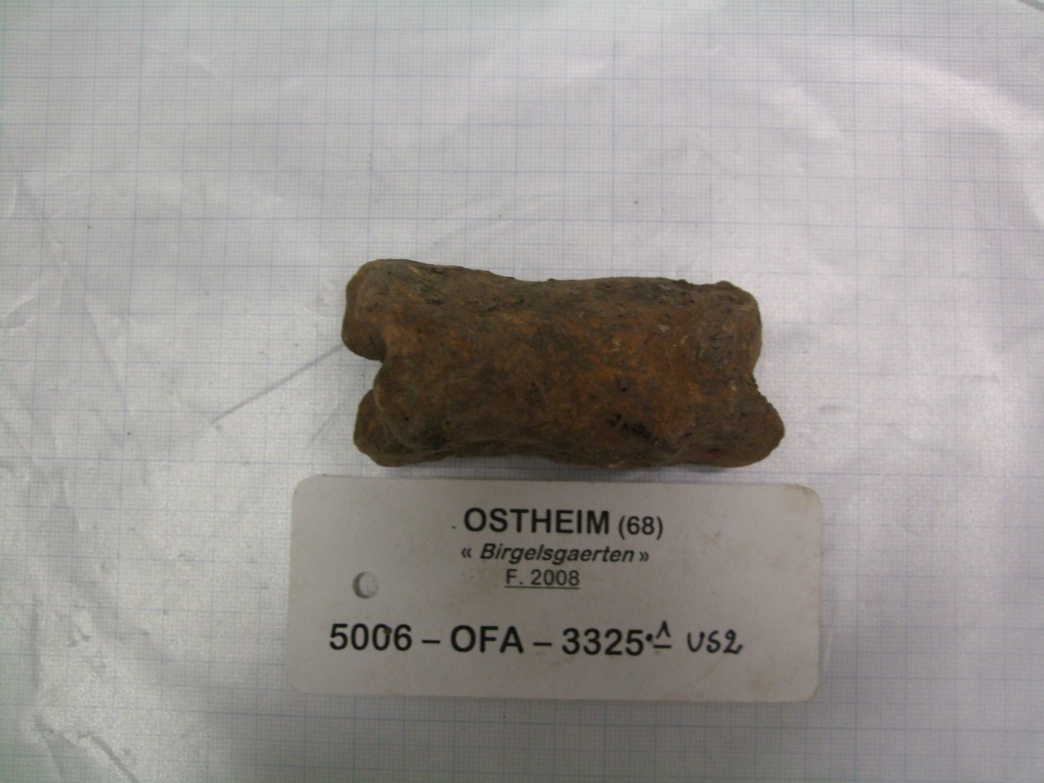 |

- 1. **Modern Caucasian bison samples** (Natalia N. Spasskarov, Institute of Zoology RAN in St. Petersburg, Russia and Zoological Museum of Lomonosov State Moscow University, Russia)

BBC27 – adult male, hunted in Kubanskaya Oblast (1895) by Great Prince Sergey Michaylowitch

BBC20 – adult individual, hunted in Kubanskaya Oblast (1911) by Great Prince Sergey Michaylowitch

BBC 24 - male, hunted in Kubanskaya Oblast (1909) by Great Prince Sergey Michaylowitch

BBC27 – female, hunted in Southern Osetia

BBC 6 – adult female, hunted in Northern Caucasus (1909) by Great Prince Sergey Michaylowitch

- 1. **Modern Lowland bison samples (**Natalia N. Spasskarov, Zoological Museum of Lomonosov State Moscow University, Russia)

Nineteen samples had been collected in Białowieża Forest, one in 1913 (Wis35) and the others in 1917, before their extinction in the wild.

# Bibliography

1. Golovanova, L.V., Hoffecker, J.F., Kharitonov, V.M. & Romanova, G.P. Mezmaiskaya Cave: A Neanderthal Occupation in the Northern Caucasus. *Current Anthropology* **40**, 77-86 (1999).

2. Baryshnikov, G., Hoffecker, J.F. & Burgess, R.L. Palaeontology and Zooarchaeology of Mezmaiskaya Cave (Northwestern Caucasus, Russia). *J. Arch. Sci.* **23**, 313-335 (1996).

3. Skinner, A.R. *et al.* ESR dating at Mezmaiskaya Cave, Russia. *Applied Radiation and Isotopes* **62**, 219-224 (2005).

4. Baryshnikov, G. Late Pleistocene brown bear (Ursus arctos) from the Caucasus. *Russian Journal of Theriology* **9**, 9-17 (2010).

5. Lioubine, V.P. *L’Acheuléen du Caucase, ERAUL 93*, (Université de Liège, 2002).

6. Baryshnikov, G.F. Pleistocene Felidae (Mammalia, Carnivora) from Paleolithic site in Kudaro caves in the Caucasus. *Proc Zool Inst RAS* **315**, 197-226 (2011).

7. Boeskorov, G.G., Bakulina, N.T., Davydov, S.P., Shchelchkova, M.V. & Solomonov, N.G. Study of Pollen and Spores from the Stomach of a Fossil Woolly Rhinoceros Found in the Lower Reaches of the Kolyma River. *Doklady Biol Sci* **436**, 23–25 (2011).

8. Bitard, B. & Madelaine, S. La grotte de La Berbie (Castels – Dordogne). *Spéléo* **AN8**, 31-40 (1994 ).

9. Madelaine, S. La Berbie (Castels). Bilan scientifique de la région aquitaine 2000. (ed. Ministère de la Culture et de la Communication, D.d.P., Sous-Direction de l’Archéologie) 24 (2001 ).

10. Merceron, G. & Madelaine, S. Molar microwear pattern and palaeoecology of ungulates from La Berbie (Dordogne, France): environmental context of the last Neandertal populations. *Boreas* **35**, 272-278 (2006 ).

11. Castel, J.-C. *et al.* La fin du Paléolithique supérieur en Quercy : l’apport de l’Igue du Gral (Sauliac-sur-Célé, Lot). in *Les sociétés du Paléolithique dans un Grand Sud-Ouest : nouveaux gisements, nouveaux résultats, nouvelles méthodes. Journées de la Société Préhistorique Française, Bordeaux, 24-25 novembre 2006*, Vol. Mémoire XLVII (eds. Jaubert, J., Ortega, I. & Bordes, J.-G.) 335-353 (Société Préhistorique Française, 2008 ).

12. Castel, J.-C., Coumont, M.-P., Boudadi-Maligne, M. & Prucca, A. Rôle et origine des grands carnivores dans les accumulations naturelles. Le cas des loups (Canis lupus) de l'Igue du Gral (Sauliac-sur-Célé, Lot, France). *Revue de Paléobiologie Genève* **29**, 411-425 (2010 ).

13. Coumont, M.-P. *et al.* Les avens-pièges à faible indice de fréquentations humaines : caractérisation paléoécologique, taphonomique et anthropologique. in *Modalité d’occupation et exploitation des milieux au Paléolithique dans le Sud-Ouest de la France : l’exemple du Quercy. Actes de la session C67, XVème Congrès mondial de l’UISPP, Lisbon, September 2006*, Vol. n° 4 (ed. Jarry, M., Brugal, J.-Ph., Ferrier C. ) 181-196 (Paleo, supplément, 2013 ).

14. Castel, J.-C. *et al.* Animal exploitation strategies in Eastern Aquitaine (France) during the Last Glacial Maximum. in *Wild Things: recent advances in Palaeolithic and Mesolithic research* (ed. F.W.F. Foulds, H.C.D., A.R. Perri, D.T.G. Clinnick, and J.W.P. Walker ) 160-174 (Oxbow Durham 2012, 2014).

15. Gamberi Almendra de Carvalho, L. *et al.* L'aven de l'Arquet - Barjac (30) : étude d'un aven piège. *Ardèche Archéologie* **28** 3-10 (2011).

16. Airvaux, J. Le site des Plumettes à Lussac-les- Châteaux (Vienne). in *Préhistoire de Poitou- Charentes*, Vol. Actes du 111é congrès des Sociétés Savantes 193-200 (CTHS, 1987).

17. Beauval, C. & Morin, E. Les repaires d’hyènes du Lussacois (Lussac-les-Châteaux,Vienne, France). Apport des sites des Plumettes et des Rochers de Villeneuve. in *Préhistoire entre Vienne et Charente. Hommes et sociétés du Paléolithique* (eds. Buisson-Catil, J. & Primault, J.) 175-190 (Association des Publications Chauvinoises, 2010).

18. Tournepiche, J.F. Les grands mammifères pléistocènes de Poitou-Charente. *Paléo* **8**, 109-141 (1996).

19. Discamps, E. Ph'D Dissertation, Hommes et hyènes face aux recompositions des communautés d’ongulés (MIS 5-3) : Eléments pour un cadre paléoécologique des sociétés du Paléolithique moyen et supérieur ancien d’Europe de l’Ouest. University of Bordeaux (2011).

20. Pétrequin, P. *Les sites littoraux néolithiques de Clairvaux-Les-Lacs et de Chalain (Jura) III Chalain station 3, 3200 - 2900 av. J.C.*, (Maison des Sciences de l'Homme, Paris, 1997).

21. Pétrequin, P., Arbogast, R.-M., Bourquin-Mignot, C., Lavier, C. & Viellet, A. Demographic growth, environmental changes and technical adaptations: responses of an agricultural community from the 32nd to the 30th centuries BC. *World Archaeology* **30**, 181-192 (1998).

22. Putelat, O. Ph'D Dissertation, Les relations homme-animal dans le monde des vivants et des morts. Étude archéozoologique des établissements et des regroupements funéraires ruraux de l’Arc jurassien et de la Plaine d’Alsace, de la fin de l’Antiquité tardive au premier Moyen Âge. University of Paris 1 Panthéon-Sorbonne (2015).
