# Supplementary figures and images for: Past climate changes, population dynamics and the origin of Bison in Europe
Source: BMC Biol. 2016 Oct 21;14:93. doi: 10.1186/s12915-016-0317-7 (PMC5075162; doi:10.1186/s12915-016-0317-7)

## Slide 1
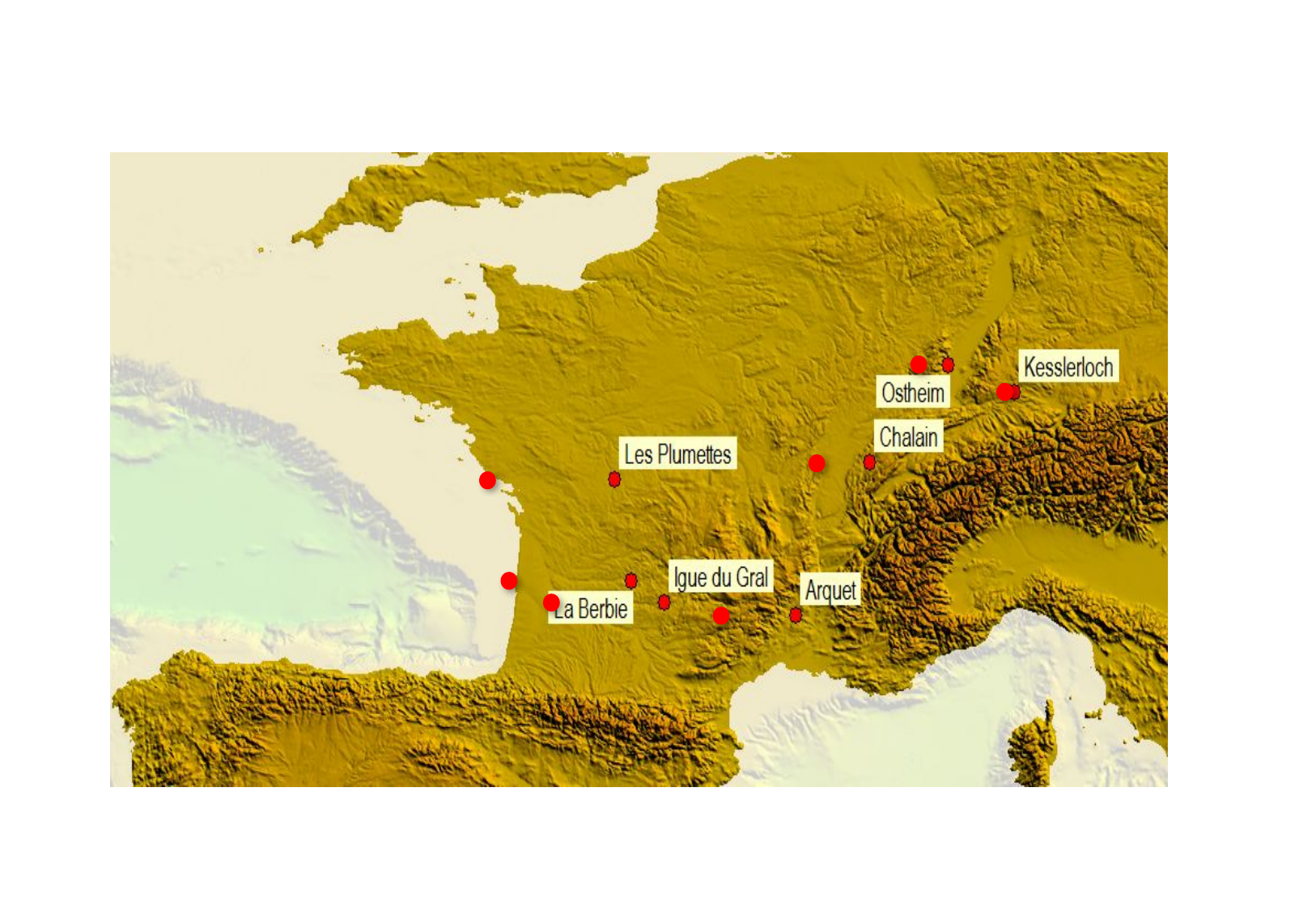

Supplement: Additional file 6: Figure S4. — Map showing the location of the various sites in Western Europe that yielded the samples described in this study. (PPTX 1296 kb) [file 12915_2016_317_MOESM6_ESM.pptx]
